# Automated fragment formula annotation for electron ionisation, high resolution mass spectrometry: application to atmospheric measurements of halocarbons

**DOI:** 10.1186/s13321-021-00544-w

**Published:** 2021-10-04

**Authors:** Myriam Guillevic, Aurore Guillevic, Martin K. Vollmer, Paul Schlauri, Matthias Hill, Lukas Emmenegger, Stefan Reimann

**Affiliations:** 1 Laboratory for Air Pollution /Environmental Technology, Empa, Swiss Federal Laboratories for Materials Science and Technology, Ueberlandstrasse 129, 8600 Dübendorf, Switzerland; 2 Université de Lorraine, CNRS, Inria, LORIA, 54000 Nancy, France

**Keywords:** Non-target screening, Automated compound identification, Combinatorics, Machine learning, Atmospheric trace gases, Electron ionisation, Time of flight mass spectrometry

## Abstract

**Background:**

Non-target screening consists in searching a sample for all present substances, suspected or unknown, with very little prior knowledge about the sample. This approach has been introduced more than a decade ago in the field of water analysis, together with dedicated compound identification tools, but is still very scarce for indoor and atmospheric trace gas measurements, despite the clear need for a better understanding of the atmospheric trace gas composition. For a systematic detection of emerging trace gases in the atmosphere, a new and powerful analytical method is gas chromatography (GC) of preconcentrated samples, followed by electron ionisation, high resolution mass spectrometry (EI-HRMS). In this work, we present data analysis tools to enable automated fragment formula annotation for unknown compounds measured by GC-EI-HRMS.

**Results:**

Based on co-eluting mass/charge fragments, we developed an innovative data analysis method to reliably reconstruct the chemical formulae of the fragments, using efficient combinatorics and graph theory. The method does not require the presence of the molecular ion, which is absent in $\sim$40% of EI spectra. Our method has been trained and validated on >50 halocarbons and hydrocarbons, with 3–20 atoms and molar masses of 30–330 g mol$^{-1}$, measured with a mass resolution of approx. 3500. For >90% of the compounds, more than 90% of the annotated fragment formulae are correct. Cases of wrong identification can be attributed to the scarcity of detected fragments per compound or the lack of isotopic constraint (no minor isotopocule detected).

**Conclusions:**

Our method enables to reconstruct most probable chemical formulae independently from spectral databases. Therefore, it demonstrates the suitability of EI-HRMS data for non-target analysis and paves the way for the identification of substances for which no EI mass spectrum is registered in databases. We illustrate the performances of our method for atmospheric trace gases and suggest that it may be well suited for many other types of samples. The L-GPL licenced Python code is released under the name ALPINAC for ALgorithmic Process for Identification of Non-targeted Atmospheric Compounds.

**Supplementary Information:**

The online version contains supplementary material available at 10.1186/s13321-021-00544-w.

## Background

Non-target screening (NTS) is an emerging approach for analysing environmental samples, with potentially revolutionary outcomes. NTS aims to detect, identify and quantify substances that are unknown in a sample, with no or very little a priori knowledge. This approach contrasts with the more established target or suspect approaches, where a sample is screened only for compounds already known or suspected to be present.

So far, NTS has been developed mostly in the fields of drinking water monitoring, food and soil analysis, forensics and metabolomics [e.g., 1–5], with human health or economic interests as the major underlying motivation. Yet, for the analysis of trace compounds in ambient or indoor air, only very limited NTS-related research has been done (e.g., the discovery of the greenhouse gas $\text {SF}_{{5}}\text {CF}_{{3}}$ [6, 7]), despite the need for a better understanding of the composition of the air. To look for emerging gases relevant for climate or air quality, suspect approaches are still nearly exclusively used [8–10].

NTS requires to measure properties that are specific for one given compound. In practice this is usually achieved by chromatographic time separation of the compounds. Further, the type of molecule ionisation and the mass range and mass accuracy are particularly relevant for NTS.

Originally, NTS was developed for medium to large molecules, therefore using soft ionisation such as chemical ionisation (CI) or electrospray ionisation (ESI), producing only a few relatively large fragments. As the molecular ion (entire molecule without one electron) is normally present and detected with soft ionisation, it is possible to reconstruct the chemical formula (i.e., the atomic assemblage, without any structural information) of the compound. To elucidate its structure, additional fragmentation and detection is required. Most (semi-)automated identification software packages were developed for CI or ESI so far [11–15] or tandem MS [16, 17].

In contrast, atmospheric trace gases consist of relatively small molecules which are best ionised by the hard electron ionisation (EI) technique. This causes a fragmentation cascade, producing many relatively small fragments; the resulting mass spectra contain valuable structural information but often lack the molecular ion [e.g., 18 Chap. 6]. Consequently, the identification of the original molecule becomes highly challenging. To circumvent this, specific instrumental source tuning may enhance the detectability of the molecular ion [19]. Alternatively, measurements could be repeated using soft ionisation, such as chemical ionisation, field desorption or field ionisation, but such a combined analytical approach is expensive and time consuming.

A well-established approach to identify a compound based on its assemblage of masses measured by EI-MS or EI-HRMS, under the absence of the molecular ion, is to perform a mass spectrum library search. Indeed, EI ionisation has been standardised already before the 90’s and produces reproducible mass spectra [e.g., 18 Chap. 5]. One of the best known EI libraries is the NIST/EPA/NIH Mass Spectral Library, with more than 250’000 experimental spectra, including approx. 140 spectra for C1 molecules [20]. However, only known and analysed compounds are present in these libraries, and identification results are therefore biased towards these compounds. Unknown emerging pollutant cannot be found by such library search.

For unknown compounds absent from spectral libraries, the identification challenge remains twofold: to identify the chemical formula of the molecular ion (also known as molecular formula annotation) and, in a second step, to identify its structure. Methods exist to identify the formula of the molecular ion in case it is present (e.g., [21]). Previous attempts have been made to predict the mass of the absent molecular ion [22] and thereby its molecular formula [23]. However, these last methods do not make use of high resolution mass data now available. Alternatively, classifiers have been developed to predict to which class(es) the unknown compound may belong [e.g., 24, 25, 26, 27].

Once candidate molecular ions have been generated, and potential classes identified, structure-generation programs (e.g., commercial software MOLGEN-MS [28, 29], open-source software OMG [30]) followed by fragmentation programs (e.g., QCEIMS [31, 32], CFM-ID [33], MetFrag [14], MOLGEN-MS [34, 35], and references therein) can be used to produce candidate mass spectra otherwise absent from libraries. In addition, when chromatographic information is available, it can be compared with retention indices if available or with a retention prediction for candidate compounds [e.g. 14, 35].

While high resolution mass spectrometers have been used for at least 30 years, and may provide sufficient information for broad, non-target screening approaches, recent developments have made this technology accessible to a larger number of laboratories. In the early 2000s, fast response, large coverage and high accuracy mass analyser, such as Orbitrap and time-of-flight (TOF) mass spectrometers, were introduced for water analysis, but only recently, first approaches have been made to use these powerful detectors also for organic atmospheric trace gases [36, 37]. Due to the challenge of identifying compounds, EI-HRMS are currently used as large mass-range coverage target or suspect screening instruments [e.g., 38, 39] but only rarely as NTS instruments. For state-of-the art EI-HRMS, there is currently a huge divide between what it can deliver in terms of sample coverage, throughput and mass resolution, compared to what identification tools can provide.

In this article, we present a workflow to reconstruct the chemical formula of fragments produced by the fragmentation of a precursor molecule in GC-EI-TOFMS. In addition, we develop a ranking method to identify most probable solutions and the reconstructed fragments that are most similar to the molecular ion. We evaluate our method by quantification of the correct results, on a training set of molecules and on an additional validation set. The entire method is written in Python and publicly available under the name ALPINAC (for ALgorithmic Process for Identification of Non-targeted Atmospheric Compounds, see "Availability of data and materials" section). While Python may not be the fastest programming language (compared to e.g. C++), it is now widely used in teaching computer science, including to students in environmental sciences and chemistry, and we hope this work will therefore be accessible to a large public.

## Experimental data

### Training data set

To develop our methodology, we use known compounds routinely measured within the Advanced Global Atmospheric Gases Experiment (AGAGE) network [40], reported in Table 1. Most of the substances are halocarbons, i.e. molecules made of a carbon chain, with halogen atoms, and are present in the atmosphere as trace gases. Structures include saturated and unsaturated chains and the presence of rings.

Within AGAGE, the chromatographic and mass spectrometric properties are obtained by measuring diluted aliquots of a pure compound [41–44] (identification at Level 1 according to the classification for non-target analysis introduced by Schymanski et al. [45]). Subsequently, an unbroken chain of calibration from the prepared synthetic primary standards to measurements on our instrument ensures that the correct compounds are measured, with the correct quantification [46].

**Table 1:** Known compounds used as training set

Compound	Chemical name	Chemical formula	Monoisotopic molecular mass Da	CAS number
$\text {C}_{{2}}\text {H}_{{6}}$	Ethane	$\text {C}_{{2}}\text {H}_{{6}}$	30.04695	74-84-0
$\text {C}_{{3}}\text {H}_{{8}}$	Propane	$\text {C}_{{3}}\text {H}_{{8}}$	44.06260	74-98-6
$\text {CH}_{{3}}$Cl	Chloromethane	$\text {CH}_{{3}}$Cl	49.99233	74-87-3
COS	Carbonyl sulphide	COS	59.96699	463-58-1
$\text {NF}_{{3}}$	Nitrogen trifluoride	$\text {NF}_{{3}}$	70.99828	7783-54-2
Benzene	Benzene	$\text {C}_{{6}}\text {H}_{{6}}$	78.04695	71-43-2
$\text {CH}_{{2}}\text {Cl}_{{2}}$	Dichloromethane	$\text {CH}_{{2}}\text {Cl}_{{2}}$	83.95336	75-09-2
HCFC-22	Chlorodifluoromethane	$\text {HCF}_{{2}}$Cl	85.97348	75-45-6
$\text {CF}_{{4}}$	Tetrafluoromethane	$\text {CF}_{{4}}$	87.99361	75-73-0
Toluene	Toluene	$\text {C}_{{7}}\text {H}_{{8}}$	92.06260	108-88-3
$\text {CH}_{{3}}$Br	Bromomethane	$\text {CH}_{{3}}$Br	93.94181	74-83-9
HCFC-142b	1-chloro-1,1-difluoroethane	$\text {H}_{{3}}\text {C}_{{2}}\text {F}_{{2}}$Cl	99.98913	75-68-3
$\text {SO}_{{2}}\text {F}_{{2}}$	Sulfuryl difluoride	$\text {SO}_{{2}}\text {F}_{{2}}$	101.95871	2699-79-8
CFC-13	Chlorotrifluoromethane	$\text {CF}_{{3}}$Cl	103.96406	75-72-9
HCFC-141b	1,1-dichloro-1-fluoroethane	$\text {H}_{{3}}\text {C}_{{2}}\text {FCl}_{{2}}$	115.95958	1717-00-6
$\text {CHCl}_{{3}}$	Chloroform	$\text {CHCl}_{{3}}$	117.91438	67-66-3
CFC-12	Dichlorodifluoromethane	$\text {CF}_{{2}}\text {Cl}_{{2}}$	119.93451	75-71-8
$\text {C}_{{2}}\text {HCl}_{{3}}$	1,1,2-trichloroethene	$\text {C}_{{2}}\text {HCl}_{{3}}$	129.91438	79-01-6
CFC-11	Trichlorofluoromethane	$\text {CFCl}_{{3}}$	135.90496	75-69-4
HCFC-124	2-chloro-1,1,1,2-tetrafluoroethane	$\text {HC}_{{2}}\text {F}_{{4}}$Cl	135.97029	2837-89-0
PFC-116	Perfluoroethane	$\text {C}_{{2}}\text {F}_{{6}}$	137.99042	76-16-4
$\text {CH}_{{3}}$I	Iodomethane	$\text {CH}_{{3}}$I	141.92795	74-88-4
$\text {SF}_{{6}}$	Sulfur hexafluoride	$\text {SF}_{{6}}$	145.96249	2551-62-4
Halon-1301	Bromo(trifluoro)methane	$\text {CF}_{{3}}$Br	147.91355	75-63-8
$\text {CCl}_{{4}}$	Tetrachloromethane	$\text {CCl}_{{4}}$	151.87541	56-23-5
CFC-115	1-chloro-1,1,2,2,2-pentafluoroethane	$\text {C}_{{2}}\text {F}_{{5}}$Cl	153.96087	76-15-3
$\text {C}_{{2}}\text {Cl}_{{4}}$	1,1,2,2-tetrachloroethene	$\text {C}_{{2}}\text {Cl}_{{4}}$	163.87541	127-18-4
Halon-1211	Bromochlorodifluoromethane	$\text {CF}_{{2}}$ClBr	163.88400	353-59-3
CFC-114	1,2-dichloro-1,1,2,2-tetrafluoroethane	$\text {C}_{{2}}\text {F}_{{4}}\text {Cl}_{{2}}$	169.93132	76-14-2
$\text {CH}_{{2}}\text {Br}_{{2}}$	Dibromomethane	$\text {CH}_{{2}}\text {Br}_{{2}}$	171.85233	74-95-3
CFC-113	1,1,2-trichloro-1,2,2-trifluoroethane	$\text {C}_{{2}}\text {F}_{{3}}\text {Cl}_{{3}}$	185.90177	76-13-1
PFC-218	Perfluoropropane	$\text {C}_{{3}}\text {F}_{{8}}$	187.98723	76-19-7
$\text {SF}_{{5}}\text {CF}_{{3}}$	Pentafluoro(trifluoromethyl)sulfur	$\text {SF}_{{5}}\text {CF}_{{3}}$	195.95930	373-80-8
PFC-c318	Octafluorocyclobutane	$\text {C}_{{4}}\text {F}_{{8}}$	199.98723	115-25-3
Halon-2402	1,2-dibromo-1,1,2,2-tetrafluoroethane	$\text {C}_{{2}}\text {F}_{{4}}\text {Br}_{{2}}$	257.83029	124-73-2
$\text {C}_{{6}}\text {F}_{{14}}$	Perfluorohexane	$\text {C}_{{6}}\text {F}_{{14}}$	337.97764	355-42-0

These 36 substances are routinely measured within the AGAGE network [40]. Identification and quantification of these compounds have been done by [41–44]. Present chemical elements are: H, C, N, O, F, S, Cl, Br and I. These are the chemical elements used as input for the knapsack algorithm. SMILES codes can be found in the Additional file 1. The corresponding latex document is the Additional file 6

### Validation data set

To validate the model after its training phase, we use a set of potentially emerging compounds, listed in Table 2. We prepared a qualitative standard containing 18 new hydrofluorocarbons (HFCs), listed under the Kigali Amendment to the Montreal Protocol [47]. The use of these substances will be progressively restricted in the coming years. Developing the capacity to check for their presence in the air, and their future molar fraction decrease, is part of supporting the application of the Kigali Amendment. The preparation of the qualitative standard is described in the Additional file 1. In addition, we use three hydrofloroolefins (HFOs) newly detected in air [8], which are replacing the HFCs in applications such as foam blowing and refrigeration [e.g., 48]: HFO-1234yf, HFO-1234ze(E) and HCFO-1233zd(E). We use already available standards prepared for these HFOs [8, 44]. Finally, we use two halogenated substances of high boiling point, which are potential emerging contaminants, which were identified and measured at Empa during a specific campaign [49].

For both the training and the validation set, the correct identification of compounds containing the monoisotopic elements fluorine and iodine may be challenging. Indeed for low abundance peaks, the absence of isotopocule may be due to a mono-isotopic element or to a non detected isotopocule, containing e.g. nitrogen or oxygen isotopes.

### Measurement by GC-EI-TOFMS

Since the 80s, specific instrumentation has been developed to tackle the challenges of measuring atmospheric halogenated trace gases: pre-concentration of the gases of interest, often present only at picomole per mole levels, chromatographic separation of substances of boiling points as low as $-128^{\circ }\text {C}$, and precise measurements to allow detection of annual trends [40, and references therein].

Our measurement system is very similar to earlier setups [43, 46]. In brief, it starts with a preconcentration trap, refrigerated at approx. $-150^{\circ }\text {C}$ using a Stirling engine, able to concentrate trace gases from up to six litres of gas (atmospheric air or reference gas mixture). Stepwise thawing of the trap eliminates the most abundant air constituents, carbon dioxide and methane, and any remaining oxygen or nitrogen, that would otherwise saturate the detector. Remaining compounds are separated by a gas chromatograph (GC), equipped with a Gaspro pre- and main column (5 m and 60 m, respectively, 0.32 mm inner diameter, Agilent), ionised and detected by a time-of-flight detector (H-TOF, Tofwerk AG, Thun, Switzerland). The detector is set to measure fragments with masses from 24 m/z (mass to charge ratio) to 300 m/z. Masses below 24 m/z are prevented from hitting the detector, to avoid saturation by potential water contamination. The mass resolution is approximately 3000 below m/z of 50 and up to 4000 above m/z of 100. The raw intensity data at each time-of-flight and each time bin are saved in a file of format hdf5 [50], which constitutes the used raw data. The total analysis time for one sample is 70 min, with 40 min of preconcentration and stepwise thawing, followed by 30 min of gas chromatography and detection by TOFMS.

Intensity data, defined as the number of ions that hit the detector at a certain time, are recorded along two axes, the time-of-flight axis (later converted to a mass axis) and the retention time (RT) axis. While the signal along the RT axis reflects the separation of molecules by the GC, the signal along the mass axis reflects the fragmentation pattern of the molecules measured by EI-MS. One fragmented, detected molecule can be visualised as a mountain ridge, producing a variety of mass peaks with various intensities, all aligned perpendicular to the time axis. First, peaks are detected and fitted along the mass axis, and afterwards along the RT axis.

Along the time-of-flight axis, at each time bin, peaks are fitted using a pseudo-Voigt function (Additional file 1). The obtained time-of-flight centres of the peaks are then converted to masses, using the mass calibration function (Additional file 1). We assume that all masses have been ionised just once. This produces a set of 20 to 30 centres of mass values with associated RT and intensity, from which the mean and standard deviation are computed, weighted by intensity. For each detected peak, the mass uncertainty ($u_{{{\,\mathrm{mass}\,}}}$) is the Euclidean distance of the calibration uncertainty ($u_{{{\,\mathrm{calibration}\,}}}$, see Additional file 1) and the measured standard deviation ($u_{{{\,\mathrm{measurement}\,}}}$):

1 $$\begin{aligned} u_{{{\,\mathrm{mass}\,}}} = 2.5 \sqrt{ u_{{{\,\mathrm{calibration}\,}}}^2 + u_{{{\,\mathrm{measurement}\,}}}^2} \end{aligned}$$

This $u_{{{\,\mathrm{mass}\,}}}$ is computed using a coverage factor of 2.5 to constrain the range of possible masses for the knapsack algorithm described below. This corresponds approximately to a 98.5% confidence interval. The resulting, expanded mass uncertainty is on average $\approx$ 6 mDa or $\approx$ 70 ppm.

Along the RT axis, data are saved with a frequency of six points per second (6 Hz). Usually, chromatography peaks last for a minimum of 4 s, producing 20–30 points per peak in the RT domain. The observed peak shape along the time axis is typical for gas chromatography and is fitted using the equation proposed by Pap et al. [51], that in our case fits well the observed tailing. When computing ratio of intensities, the obtained isotopic pattern accuracy ranges from 1 to 5 %, depending on peak intensities, and is on average 2.0 %. This value mostly reflects the precision of the entire measurement system. Finally, co-eluting mass peaks are grouped together.

Routine quality control of instrumental performance includes measuring blanks to check for potential contaminants coming from the measurement setup itself, drifts in retention time due to column ageing or water contamination, and stability of intensity ratios of mass fragments belonging to the same compound.

## Method for automated fragment formula identification

### Method overview

The output after peak fitting and mass calibration is a dataset of mass/charge ratio (*m*/*z*), each with intensity (in *V*) and uncertainty (in ppm), at a precise retention time (in seconds). Co-eluting peaks may correspond to chemical fragments of a unique molecule, or a small number of distinct molecules. They are therefore grouped into one time slot of approx. 2 s width to be treated together by the identification algorithm. We consider each time slot separately.

**Fig. 1:**
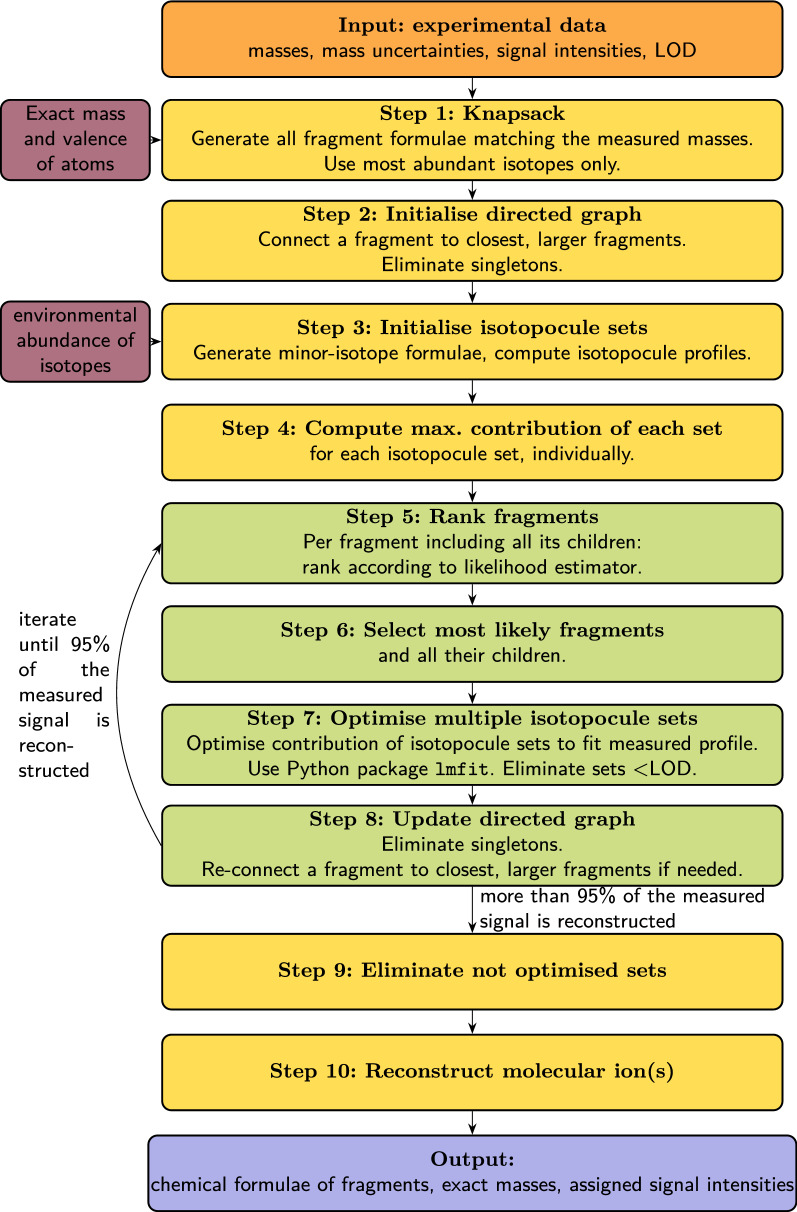
Overview of the method for automated identification of fragment formulae. Orange box (top): input measured data. Two mauve boxes (left): input chemical data [56]. Yellow boxes are steps done just once. Steps 1 to 4: steps of initialisation. Green boxes, steps 5 to 8: steps repeated until a certain fraction of the measured signal has been reconstructed, here 95%. Steps 9 and 10, yellow box: final steps, done just once. Blue box: output data, list of most likely fragments together with associated likelihood and ranking

The overview of our method is depicted in Fig. 1. The general approach is to consider separately each group of co-eluting fragments, and to reconstruct the chemical formula of each fragment based on two types of information:

- From the experimental data produced by GC-EI-TOF analysis, we use the measured mass and measured signal intensity of each peak. For the measured masses, the uncertainty (Eq. (1)) is computed following metrology principles (Fig. 1, yellow box **Input**). It is known that using mass information only is not enough to correctly reconstruct chemical formulae, even at <1 ppm accuracy [52];
- Therefore, we combine these experimental data with chemical information that is universal, i.e. true for any given molecule: exact mass and valence of chemical elements, known environmental stable isotopic abundances (Fig. 1, two mauve boxes).

**Fig. 2:**
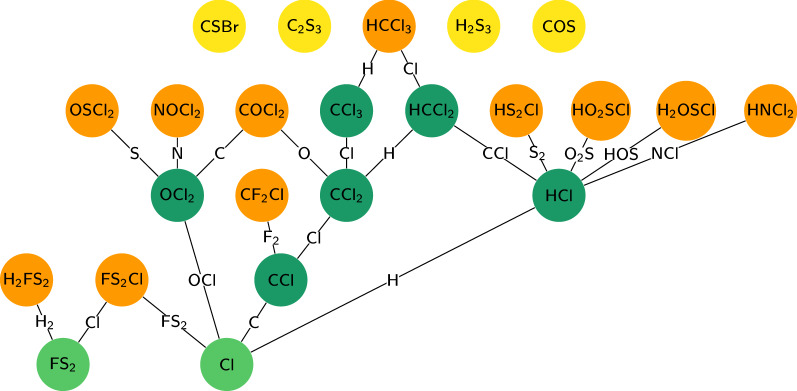
Directed acyclic pseudo-fragmentation graph obtained in Step 2, with all the candidate fragments (nodes) from the knapsack algorithm for $\text {CCl}_{{4}}$. One observes 23 nodes, with 2 leaves (or smallest possible fragments, in light green), 15 maximal fragments (in orange and yellow), of which 4 have no children and are therefore singletons (in yellow). The latter are eliminated in Step 2

In practice, the identification method combines algorithms for two purposes: (i) algorithms that enumerate solutions in an exhaustive way, according to given constrains (Fig. 1, steps 1 and 3); (ii) algorithms that eliminate unlikely solutions, based on other constraints (Fig. 1, steps 2, 7, 8 and 9).

The developed workflow is as follows (steps are numbered as in Fig. 1):

Step 1: To start, possible atom assemblages matching the measured masses, within uncertainty, are exhaustively generated. This step usually produces a large number of possible chemical formulae;
Step 2: all generated formulae are organised in a pseudo-fragmentation graph. This step relies on the specificity of EI-MS, producing many various fragments from the same precursor ion molecule. The irrelevant or unlikely formulae are discarded;
Step 3: isotopocules (i.e. molecules having the same type and number of atoms, but where at least one atom is a different isotope) are generated;
Step 4: for each set of isotopocules, the isotopic pattern of fragments (theoretical intensity profile) along the mass axis is generated. The optimum contribution to the measured profile, of each set separately, is computed;
Step 5: using a specifically designed likelihood estimator, all candidate chemical formulae are set out in order of preference, number one being the most likely, and the last one being the least likely;
Step 6: This ordering enables the selection of the most likely candidate(s);
Step 7: the intensity of each isotopocule set is optimised by comparison to the measured mass profile, using a machine learning technique. This is by far the computationally most costly step. Isotopocule series characterised by a total intensity below a given threshold, usually, the instrumental limit of detection (LOD), are eliminated.
Step 8: The pseudo-fragmentation tree is updated, and the optimisation procedure resumes at Step 5, until a predefined fraction of the measured signal is reproduced.
Step 9: All remaining, not optimised candidate chemical formulae are deleted. The remaining candidates constitute the final list of most likely correct chemical formulae. Each measured mass may have zero, one or several assigned chemical formulae.
Step 10: The last step is a tentative reconstruction of the molecular ion(s). Most likely molecular ion(s) are generated based on the largest fragments from the graph and they are set out in order according to their computed likelihood value.

Each step of the method is explained in details hereafter. In the Additional file 1, Sect. 6, we give a numerical example with the mass spectrum that will turn out to correspond to $\text {CCl}_{{4}}$.

### Step 1: Generating fragment formulae: the knapsack algorithm

The aim of the *knapsack step* is to recover all chemical formulae that could correspond to each detected fragment, given its mass and uncertainty, and excluding all other chemical formulae that would not fulfil the criteria of measured mass and uncertainty. This knapsack step produces the correct chemical formulae, usually along with many other incorrect formulae. The aim of subsequent steps will be to eliminate the incorrect formulae using additional constrains.

We use combinatorics to generate the chemical formulae of candidate fragments, for each mass detected in a spectrum. In particular, we develop a variant of the knapsack algorithm [53, 54], dedicated to our setting, which will be described below.

#### The knapsack algorithm in combinatorics

Combinatorics are mathematical algorithms of fast and exhaustive enumeration, and the knapsack problem is a well-known topic in this area (see e.g. the handbook on algorithms [55]). The problem is usually stated as follows: given a knapsack of maximum available capacity (e.g. mass), and a set of items each of a specific capacity, find subset(s) of items that can fit into the knapsack, while optimising some other quantity (usually maximizing the total price of the items). In our setting, the knapsack is a fragment of given mass (within the uncertainty range), and the items are atoms of exact given mass. We are interested in enumerating all possible combinations of atoms so that the sum of their masses fits the measured fragment mass within the uncertainty margin. An unbounded number of each atom is available i.e. each atom type can be used multiple times, this is also known as the *unbounded* knapsack problem. Contrary to the classical problem in combinatorics, we do not optimise another parameter. Instead, we are interested in listing all possibilities. In this work, we design an algorithm for a fast and exhaustive enumeration of all the solutions to the knapsack problem.

The inputs of our dedicated knapsack algorithm are the measured masses of the fragments with uncertainties, and a list of masses of elements that are expected to form the fragments. Because the exhaustive list of solutions grows exponentially with the number of elements, we will introduce different techniques to avoid as early as possible enumerating wrong chemical formulae, while still being exhaustive.

#### Targeted mass with uncertainty

This section describes the basic algorithm to enumerate all the possibilities. The target mass, which is one measured mass, is denoted by *m*; the set of item masses, which are the exact masses of chemical elements (IUPAC: [56]), is made of *I* distinct positive values $m_i$, labelled $m_0$ to $m_{I-1}$, sorted in increasing order, that is, $m_i < m_{i+1}$ for all *i*. We do not consider uncertainties of the atomic masses, which are negligible compared to the TOF analytical mass uncertainties. A solution of a knapsack problem is encoded as a vector of positive integers $[a_0,a_1,\ldots ,a_{I-1}]$ where $a_i$ is the number of items of mass $m_i$; it is 0 if the item *i* is not in the solution. Algorithms 1, 2 (in pseudo-code) describe the basic recursive enumeration. An iterative (non-recursive) function is also possible but we implemented a recursive function. 
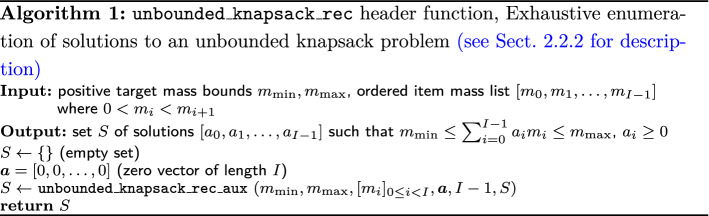

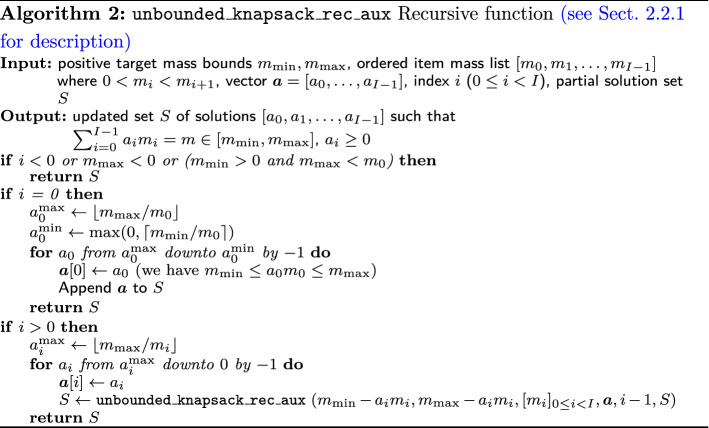




**Fig. 3:**
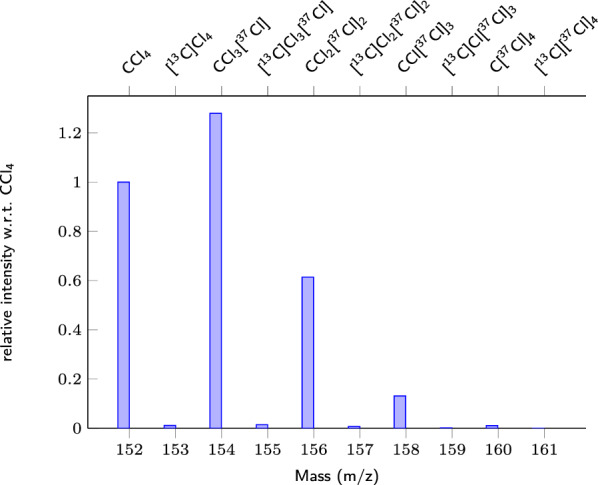
isotopocules of $\text {CCl}_{{4}}$ with mass and relative intensity w.r.t. $\text {CCl}_{{4}}$. The abundant formula $\text {CCl}_{{4}}$ has one isotopocule $\text {CCl}_{{3}}$[$^{37}$Cl] of relative intensity greater than one (1.279504). See Table S8 in the Additional file 1 for numerical data

**Table 2:** Known compounds used as validation set

Compound	Chemical name	Chemical formula	Monoisotopic molecular mass Da	CAS number
Kigali Amendment to the Montreal Protocol				
HFC-41	fluoromethane	$\text {CH}_{{3}}$F	34.021878	593-53-3
HFC-32	difluoromethane	$\text {CH}_{{2}}\text {F}_{{2}}$	52.012456	75-10-5
HFC-152	1,2-difluoroethane	$\text {C}_{{2}}\text {H}_{{4}}\text {F}_{{2}}$	66.028106	624-72-6
HFC-152a	1,1-difluoroethane	$\text {C}_{{2}}\text {H}_{{4}}\text {F}_{{2}}$	66.028106	75-37-6
HFC-23	fluoroform	$\text {CHF}_{{3}}$	70.003035	75-46-7
HFC-143	1,1,2-trifluoroethane	$\text {C}_{{2}}\text {H}_{{3}}\text {F}_{{3}}$	84.018685	430-66-0
HFC-143a	1,1,1-trifluoroethane	$\text {C}_{{2}}\text {H}_{{3}}\text {F}_{{3}}$	84.018685	420-46-2
HFC-134	1,1,2,2-tetrafluoroethane	$\text {C}_{{2}}\text {H}_{{2}}\text {F}_{{4}}$	102.009263	359-35-3
HFC-134a	1,1,1,2-tetrafluoroethane	$\text {C}_{{2}}\text {H}_{{2}}\text {F}_{{4}}$	102.009263	811-97-2
HFC-125	pentafluoroethane	$\text {C}_{{2}}\text {HF}_{{5}}$	119.999841	354-33-6
HFC-245ca	1,1,2,2,3-pentafluoropropane	$\text {C}_{{3}}\text {H}_{{3}}\text {F}_{{5}}$	134.015491	679-86-7
HFC-245fa	1,1,1,3,3-pentafluoropropane	$\text {C}_{{3}}\text {H}_{{3}}\text {F}_{{5}}$	134.015491	460-73-1
HFC-365mfc	1,1,1,3,3-pentafluorobutane	$\text {C}_{{4}}\text {H}_{{5}}\text {F}_{{5}}$	148.031141	406-58-6
HFC-236cb	1,1,1,2,2,3-hexafluoropropane	$\text {C}_{{3}}\text {H}_{{2}}\text {F}_{{6}}$	152.006069	677-56-5
HFC-236ea	1,1,1,2,3,3-hexafluoropropane	$\text {C}_{{3}}\text {H}_{{2}}\text {F}_{{6}}$	152.006069	431-63-0
HFC-236fa	1,1,1,3,3,3-hexafluoropropane	$\text {C}_{{3}}\text {H}_{{2}}\text {F}_{{6}}$	152.006069	690-39-1
HFC-227ea	1,1,1,2,3,3,3-heptafluoropropane	$\text {C}_{{3}}\text {HF}_{{7}}$	169.996647	431-89-0
HFC-43-10mee	1,1,1,2,2,3,4,5,5,5-decafluoropentane	$\text {C}_{{5}}\text {H}_{{2}}\text {F}_{{10}}$	251.999682	138495-42-8
HFOs				
HFO-1234yf	2,3,3,3-tetrafluoroprop-1-ene	$\text {H}_{{2}}\text {C}_{{3}}\text {F}_{{4}}$	114.009263	754-12-1
HFO-1234ze(E)	(E)-1,3,3,3-tetrafluoroprop-1-ene	$\text {H}_{{2}}\text {C}_{{3}}\text {F}_{{4}}$	114.009263	29118-24-9
HCFO-1233zd(E)	(E)-1-chloro-3,3,3-trifluoro prop-1-ene	$\text {H}_{{2}}\text {C}_{{3}}\text {F}_{{3}}$Cl	129.979712	102687-65-0
Halogenated compounds with high boiling point				
HCBD	1,1,2,3,4,4-hexachlorobuta-1,3-diene	$\text {C}_{{4}}\text {Cl}_{{6}}$	257.813116	87-68-3
TCHFB	1,2,3,4-tetrachlorohexafluorobutane	$\text {C}_{{4}}\text {Cl}_{{4}}\text {F}_{{6}}$	301.865830	375-45-1

Identification and quantification of these 23 compounds has been done by [8, 44, 49, 70]. Present chemical elements: H, C, N, O, F, Cl. Chemical elements used as input for the knapsack algorithm are the same as for the training set: H, C, N, O, F, S, Cl, Br and I. SMILES codes can be found in the Additional file 1

**Fig. 4:**
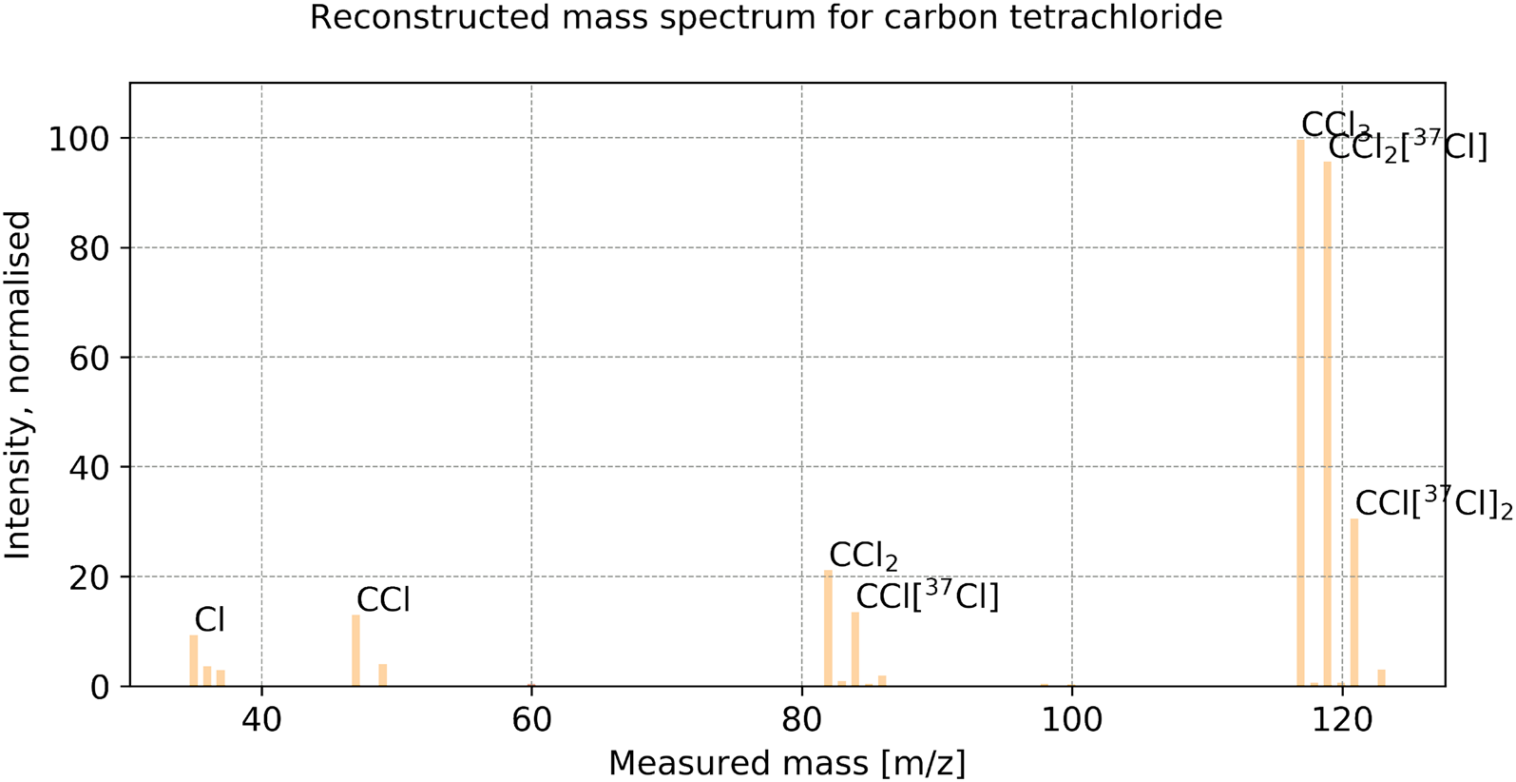
Reconstructed mass spectrum for $\text {CCl}_{{4}}$, when setting as target that 95% of the signal should be reconstructed. Numerical values can be found in the Additional file 3

#### Only the most abundant isotope of each element used as input

We consider nine elements (H, C, N, O, F, S, Cl, Br, I) with their stable isotopes (if any), making a list of 19 different exact masses^1^ that can be combined to form a molecule (we omit the elements that are rarely found in volatile atmospheric trace gases, such as Si, P and metals^2^). The electron ionisation fragmentation produces isotopocule fragments. For example for the molecule $\text {CCl}_{{4}}$, we may observe $\text {CCl}_{{4}}$ made of only abundant isotopes ($^{12}$C or C in short notation, $^{35}$Cl or Cl in short notation), and isotopocules containing $^{13}$C and $^{37}$Cl (see the complete list of isotopocules provided in Fig. 3 and in the Additional file 1: Table S8).

To reduce the enumeration of the knapsack, the input is limited to the mass of the most abundant isotope of each element (e.g.  C of mass 12.000000 g mol$^{-1}$ for carbon, Cl of mass 34.96885271 g mol$^{-1}$ for chlorine), making a list of 9 exact masses to be used for the enumeration, instead of 19. For relatively small molecules, the fragment made of only abundant isotopes has usually the highest intensity (or second highest, Fig. 3), hence producing a much smaller mass uncertainty than for the other isotopocules. The target mass range is thinner, reducing the knapsack enumeration. By doing so, we obtain possible solutions with the knapsack only for some of the most abundant fragments. Once a fragment made of abundant isotopes is generated by the knapsack, we later enumerate all its isotopocules containing minor isotopes, in Step 3 (Fig. 1). This is further explained in "Step 4: Computing the optimum contribution for each isotopocule set individually" section.

#### Optimisation of the knapsack enumeration: double bond equivalent (DBE) criterion with meet-in-the-middle optimisation algorithm

Not all sets of atoms form a valid chemical formula. Indeed, each atom allows a maximum number of chemical bonds with other atoms, according to its valence. This can be expressed using the *double bond equivalent* (DBE), or sum of number of rings and double bonds in a given chemical formula. The DBE is computed with [§6.4.4 Eq. (6.9) 18]

2 $$\begin{aligned} {{\,\mathrm{DBE}\,}}= 1 + 0.5 \times \sum _{i}^{i_{\max }}N_i(V_i-2) \end{aligned}$$

where $V_i$ is the valence of element *i* and $N_i$ is the number of atoms of element *i* in the chemical formula. For EI-MS, since we expect no cluster formation in the ionisation source, the minimum valence for a chemical formula is 0. We do not set any maximum valence value. For the sulphur element, where multiple valences are possible, we chose the maximum value (6), according to one of the golden rules for identification [57].

Of all chemical formulae matching the considered mass domain, only a fraction are chemically valid. This means that to reduce the enumeration time of the knapsack, one strategy is to avoid enumerating chemical formulae with a negative DBE value. We explain hereafter how we implement this.

In the early 80’s, cryptologists^3^ formulated a meet-in-the-middle strategy to speed-up the enumeration of all solutions of a knapsack problem [53]. The key-ingredient is to partition the candidate items in two sets. Applying this strategy to our topic, one enumerates all possibilities made of items of the first set and whose mass is smaller or equal to the target mass. The possibilities are ordered by increasing mass. Meanwhile, one does the same with the items of the second set. The two sets are chosen so that the respective running-time of the two enumerations are balanced, in order to minimize the total running-time. We end up with two lists of masses in increasing order, of value between 0 and the target mass. Then reading onward the first list and downwards the second list, one looks for pairs of partial solutions, one from each list, so that the paired mass matches the target mass. A numerical example is given in the Additional file 1 in Sect. 6.1.

We adapt this strategy to speed up the solution of our problem. We partition the input atoms in two sets: the set of multivalent atoms (C, N, O, S) and the set of monovalent atoms (H, F, Cl, Br, I). First, all solutions made of multivalent atoms, and smaller or equal to the target mass, are generated. To a generated multivalent-atom-solution, a maximum number of monovalent atoms, twice the DBE value (cf. Eq. (2)), can be added and still form a valid chemical formula. In this way, the partial solutions made of multivalent atoms give us an upper bound on the number of monovalent atoms that can complete the fragment, reducing considerably the enumeration of partial possibilities with monovalent atoms. In particular, it gives an upper bound on the number of hydrogen H. A numerical example is given in the Additional file 1 in Sect. 6.2.

The list made of multivalent atoms is precomputed for the heaviest mass first, and can be re-used for the smaller masses. We also implemented a way to re-use the list of monovalent atoms precomputed for the heaviest target mass, when processing the lighter target masses. Our strategy turned out to be fast enough for the considered mass ranges (see runtime in "How wrong knapsack solutions are rejected and implications for computation runtime" section) and we did not investigate further optimisations. Dührkop et al. [58] have a very different approach well-suited for molecular masses of around 1000 Da, implemented in the SIRIUS software suite for tandem MS [59].

After Step 1 (Fig. 1), for each measured mass, all chemical formulae that are in agreement with the measured mass within its uncertainty, made of abundant isotopes, and having a positive DBE value, are generated. At this stage, the fragment formulae are not uniquely identified by the knapsack: for each measured mass there are either too many possibilities, or none (usually because the fragment may contain non-abundant isotopes).

### Step 2: Organisation of the results in a pseudo-fragmentation graph

The aim of Step 2 is to organise all chemical formulae generated in Step 1 according to a specific order, to help identify and delete unlikely chemical formulae.

With EI-MS, a fragmentation cascade happens due to the high ionisation energy, i.e. several fragmentation steps one after the other [e.g., 18]. A fragmentation step produces an ionised fragment (detected) and a neutral (not detected). Each detected fragment may result from one or several fragmentation steps. As the EI fragmentation takes place under vacuum with pressure usually below $10^{-5}$ bar, we do not expect to see clusters originating from agglomeration of (fragments of) the molecular ion with other chemical species. On the contrary, all detected fragments are pieces of the original molecule. If all fragmentation steps are known, one can organise the fragments in an acyclic directed graph (Fig. 2). The nodes are the fragments. One edge is one fragmentation step. This forms a *fragmentation graph*. Potentially, several fragmentation pathways in the EI source may produce the same end fragment(s). But thanks to directions, the graph is acyclic.

We organise all the candidate formulae from the knapsack in a directed graph (with the class DiGraph provided in the Python package Networkx, [60]). Each node on the graph is a candidate fragment, with associated attributes, such as its chemical formula, its exact mass, the associated measured mass(es), and the list of minor isotopocules that will be generated at the next step. An edge is set from a node to another if the chemical formula of the first fragment admits the chemical formula of the second one as subformula (e.g. $\text {CCl}_{{3}}$ has subfragment CCl, see Fig. 2 presenting the directed graph obtained with all knapsack solutions of $\text {CCl}_{{4}}$). This mimics the possible fragmentation pathways. In other words, we define a *partial* ordering of the fragments (it is not *total* because, for example, there is no relation between candidate fragments $\text {CCl}_{{3}}$ and CSBr, cf. Fig. 2). The *maximal fragments*^4^ have no ancestor but may have children (Fig. 2, nodes in orange and yellow), they are the maximal elements of the ensemble of fragments. If the molecular ion is present, it is one of the maximal fragments. As with EI, the molecular ion is often absent (as for 14 compounds of the training set, see Table 3), several maximal fragments are allowed. Also, to account for potential co-elution of different molecules, several connected components are allowed. This algorithm does not use any structural information, only the candidate chemical formulae, producing only a pseudo-fragmentation graph, not a chemically realistic one, contrary to previous algorithms [21]. We do not use a list of expected neutral losses (as in e.g., [12]) due to the high heterogeneity of our chosen molecules. Therefore, it is likely that some edges are actually not structurally possible, but this is not relevant at this stage. Optimisation strategies for an efficient construction of this directed graph are reported in the Additional file 1 (Sect. 5.1).

**Table 3:** Known compounds used as training set: presence of the molecular ion

Compound	Chemical formula	Molecular ion present	Reconstructed mol. ion
$\text {C}_{{2}}\text {H}_{{6}}$	$\text {C}_{{2}}\text {H}_{{6}}$	Yes	1
$\text {C}_{{3}}\text {H}_{{8}}$	$\text {C}_{{3}}\text {H}_{{8}}$	Yes	1
$\text {CH}_{{3}}$Cl	$\text {CH}_{{3}}$Cl	Yes	1
COS	COS	Yes	1
$\text {NF}_{{3}}$	$\text {NF}_{{3}}$	Yes	1
Benzene	$\text {C}_{{6}}\text {H}_{{6}}$	Yes	1
$\text {CH}_{{2}}\text {Cl}_{{2}}$	$\text {CH}_{{2}}\text {Cl}_{{2}}$	yes	1
HCFC-22	$\text {HCF}_{{2}}$Cl	Yes	1
$\text {CF}_{{4}}$	$\text {CF}_{{4}}$	$\text {CF}_{{3}}$	none
Toluene	$\text {C}_{{7}}\text {H}_{{8}}$	Yes	1
$\text {CH}_{{3}}$Br	$\text {CH}_{{3}}$Br	Yes	1
HCFC-142b	$\text {H}_{{3}}\text {C}_{{2}}\text {F}_{{2}}$Cl	$\text {H}_{{2}}\text {C}_{{2}}\text {F}_{{2}}$Cl, $\text {H}_{{3}}\text {C}_{{2}}$FCl, $\text {H}_{{3}}\text {C}_{{2}}\text {F}_{{2}}$	1
$\text {SO}_{{2}}\text {F}_{{2}}$	$\text {SO}_{{2}}\text {F}_{{2}}$	Yes	O3FCl
CFC-13	$\text {CF}_{{3}}$Cl	$\text {CF}_{{2}}$Cl, $\text {CF}_{{3}}$	1
HCFC-141b	$\text {H}_{{3}}\text {C}_{{2}}\text {FCl}_{{2}}$	$\text {H}_{{2}}\text {C}_{{2}}\text {Cl}_{{2}}$, $\text {H}_{{3}}\text {C}_{{2}}$FCl	2 (1: $\text {C}_{{2}}\text {H}_{{4}}$FCl)
$\text {CHCl}_{{3}}$	$\text {CHCl}_{{3}}$	Yes	1
CFC-12	$\text {CF}_{{2}}\text {Cl}_{{2}}$	Yes	1
$\text {C}_{{2}}\text {HCl}_{{3}}$	$\text {C}_{{2}}\text {HCl}_{{3}}$	Yes	1
CFC-11	$\text {CFCl}_{{3}}$	Yes	1
HCFC-124	$\text {HC}_{{2}}\text {F}_{{4}}$Cl	Yes	1
PFC-116	$\text {C}_{{2}}\text {F}_{{6}}$	$\text {C}_{{2}}\text {F}_{{5}}$	1
$\text {CH}_{{3}}$I	$\text {CH}_{{3}}$I	Yes	1
$\text {SF}_{{6}}$	$\text {SF}_{{6}}$	$\text {SF}_{{5}}$	1
Halon-1301	$\text {CF}_{{3}}$Br	Yes	1
$\text {CCl}_{{4}}$	$\text {CCl}_{{4}}$	$\text {CCl}_{{3}}$	2 (1: $\text {CHCl}_{{3}}$)
CFC-115	$\text {C}_{{2}}\text {F}_{{5}}$Cl	$\text {C}_{{2}}\text {F}_{{4}}$Cl, $\text {C}_{{2}}\text {F}_{{5}}$	1
$\text {CCl}_{{2}}$=$\text {CCl}_{{2}}$	$\text {C}_{{2}}\text {Cl}_{{4}}$	Yes	1
Halon-1211	$\text {CF}_{{2}}$ClBr	CFClBr, $\text {CF}_{{2}}$Br, $\text {CF}_{{2}}$Cl	1
CFC-114	$\text {C}_{{2}}\text {F}_{{4}}\text {Cl}_{{2}}$	$\text {C}_{{2}}\text {F}_{{3}}\text {Cl}_{{2}}$, $\text {C}_{{2}}\text {F}_{{4}}$Cl	1
$\text {CH}_{{2}}\text {Br}_{{2}}$	$\text {CH}_{{2}}\text {Br}_{{2}}$	Yes	1
CFC-113	$\text {C}_{{2}}\text {F}_{{3}}\text {Cl}_{{3}}$	Yes	1
PFC-218	$\text {C}_{{3}}\text {F}_{{8}}$	$\text {C}_{{3}}\text {F}_{{7}}$	1
$\text {SF}_{{5}}\text {CF}_{{3}}$	$\text {SF}_{{5}}\text {CF}_{{3}}$	$\text {SF}_{{5}}$, $\text {CF}_{{3}}$	$\text {SF}_{{5}}$, $\text {CF}_{{4}}$
PFC-c318	$\text {C}_{{4}}\text {F}_{{8}}$	$\text {C}_{{3}}\text {F}_{{5}}$	$\text {C}_{{3}}\text {F}_{{6}}$
Halon-2402	$\text {C}_{{2}}\text {F}_{{4}}\text {Br}_{{2}}$	Yes	1
$\text {C}_{{6}}\text {F}_{{14}}$	$\text {C}_{{6}}\text {F}_{{14}}$	$\text {C}_{{5}}\text {F}_{{9}}$	$\text {C}_{{4}}\text {F}_{{10}}$

If the molecular ion is absent, we give the detected maximal fragments instead. Note that several maximal fragments may be detected for one substance. The last column indicates the ranking of the correct molecular ion, if reconstructed by our method, or which molecular ion(s) is (are) reconstructed (if any)

From this pseudo-fragmentation graph, we would like to eliminate the isolated nodes, or nodes not being connected to any other node (singletons), with neither ancestors nor children (Fig. 2, nodes in yellow). They may correspond to (i) impurities produced for example by GC bleed or (ii) solutions from the knapsack that are unlikely, in particular with an atom absent from all other nodes. But we need to account for very small molecules such as CFC-11 ($\text {CFCl}_{{3}}$) or CFC-13 ($\text {CF}_{{3}}$Cl) that produce a very limited number of different fragments, without the molecular ion: if one measured mass has only singletons as candidate fragments, we do not eliminate them.

At the end of Step 2, usually a few unlikely knapsack solutions being singletons have been eliminated.

### Step 3: Enumeration of chemical formulae containing minor isotopes

The knapsack algorithm produces candidate chemical formulae made of abundant isotopes only. But all isotopocules of a fragment are expected to be present in a given time slot (the very rare ones which are below the detection limit of the mass spectrometer may not be detected). Therefore, for each candidate chemical formula, we now generate a set of all isotopocules including their relative intensities based on their natural isotopic abundances [56]).

Hereafter, we name *knapsack formula* a chemical formula from the knapsack, and *minor-isotope formula* a chemical formula with at least one minor isotope, even if this isotopocule is expected to be more abundant than the *abundant chemical formula*. For example, $\text {CCl}_{{4}}$ is called knapsack formula, while $\text {CCl}_{{3}}$[$^{37}$Cl] is called minor-isotope formula.

For each possible knapsack formula, we generate the list of all possible isotopocules. Again, this is an enumeration process using combinatorics. If the chemical formula is made of atoms that are monoisotopic, the list contains the knapsack formula only, whose intensity is one, that is, 100%. Otherwise, for each minor-isotope formula, we compute its exact mass and expected intensity relative to the knapsack formula [56, 61]. The isotopocules of the knapsack formula $\text {CCl}_{{4}}$ with their relative intensities are shown in Fig. 3 and the corresponding numerical values can be found in Table S8 of the Additional file 1.

**Table 4:** Known compounds used as validation set: presence of the molecular ion

Compound	Chemical formula	Molecular ion present	Reconstructed mol. ion
Kigali Amendment to the Montreal Protocol			
HFC-41	$\text {CH}_{{3}}$F	Yes	1
HFC-32	$\text {CH}_{{2}}\text {F}_{{2}}$	Yes	1
HFC-152	$\text {C}_{{2}}\text {H}_{{4}}\text {F}_{{2}}$	Yes	1
HFC-152a	$\text {C}_{{2}}\text {H}_{{4}}\text {F}_{{2}}$	Yes	1
HFC-23	$\text {CHF}_{{3}}$	$\text {CF}_{{3}}$, $\text {HCF}_{{2}}$	1
HFC-143	$\text {C}_{{2}}\text {H}_{{3}}\text {F}_{{3}}$	Yes	1
HFC-143a	$\text {C}_{{2}}\text {H}_{{3}}\text {F}_{{3}}$	Yes	1
HFC-134	$\text {C}_{{2}}\text {H}_{{2}}\text {F}_{{4}}$	Yes	1
HFC-134a	$\text {C}_{{2}}\text {H}_{{2}}\text {F}_{{4}}$	Yes	1
HFC-125	$\text {C}_{{2}}\text {HF}_{{5}}$	$\text {C}_{{2}}\text {F}_{{5}}$, $\text {HC}_{{2}}\text {F}_{{4}}$	1
HFC-245ca	$\text {C}_{{3}}\text {H}_{{3}}\text {F}_{{5}}$	$\text {C}_{{3}}\text {H}_{{2}}\text {F}_{{3}}$, $\text {C}_{{2}}\text {H}_{{3}}\text {F}_{{2}}$, $\text {C}_{{3}}\text {HF}_{{4}}$	$\text {C}_{{3}}\text {H}_{{2}}\text {F}_{{4}}$ (1), $\text {C}_{{3}}\text {H}_{{3}}\text {F}_{{3}}$ (2)
HFC-245fa	$\text {C}_{{3}}\text {H}_{{3}}\text {F}_{{5}}$	Yes	1
HFC-365mfc	$\text {C}_{{4}}\text {H}_{{5}}\text {F}_{{5}}$	$\text {C}_{{4}}\text {H}_{{5}}\text {F}_{{4}}$, $\text {C}_{{3}}\text {H}_{{2}}\text {F}_{{5}}$	$\text {C}_{{4}}\text {H}_{{6}}\text {F}_{{4}}$ (1), $\text {C}_{{3}}\text {H}_{{3}}\text {F}_{{5}}$ (2)
HFC-236cb	$\text {C}_{{3}}\text {H}_{{2}}\text {F}_{{6}}$	$\text {C}_{{3}}\text {H}_{{2}}\text {F}_{{5}}$, $\text {C}_{{3}}\text {HF}_{{6}}$	1
HFC-236ea	$\text {C}_{{3}}\text {H}_{{2}}\text {F}_{{6}}$	$\text {C}_{{3}}\text {H}_{{2}}\text {F}_{{5}}$	1
HFC-236fa	$\text {C}_{{3}}\text {H}_{{2}}\text {F}_{{6}}$	$\text {C}_{{3}}\text {H}_{{2}}\text {F}_{{5}}$	1
HFC-227ea	$\text {C}_{{3}}\text {HF}_{{7}}$	$\text {C}_{{3}}\text {HF}_{{6}}$	1
HFC-43-10mee	$\text {C}_{{5}}\text {H}_{{2}}\text {F}_{{10}}$	$\text {C}_{{4}}\text {H}_{{2}}\text {F}_{{7}}$, $\text {C}_{{5}}$HF8, $\text {C}_{{5}}\text {H}_{{2}}$	$\text {C}_{{4}}\text {H}_{{2}}\text {F}_{{8}}$ (1), $\text {C}_{{4}}\text {H}_{{3}}\text {F}_{{7}}$ (2)
HFOs			
HFO-1234yf	$\text {C}_{{3}}\text {H}_{{2}}\text {F}_{{4}}$	yes	1
HFO-1234ze(E)	$\text {C}_{{3}}\text {H}_{{2}}\text {F}_{{4}}$	Yes	1
HCFO-1233zd(E)	$\text {C}_{{3}}\text {H}_{{2}}\text {ClF}_{{3}}$	Yes	1
Halogenated compounds with high boiling point			
HCBD	$\text {C}_{{4}}\text {Cl}_{{6}}$	Yes	1
TCHFB	$\text {C}_{{4}}\text {Cl}_{{4}}\text {F}_{{6}}$	$\text {C}_{{4}}\text {ClF}_{{6}}$, $\text {C}_{{3}}\text {Cl}_{{3}}\text {F}_{{4}}$	$\text {C}_{{3}}\text {Cl}_{{3}}\text {F}_{{5}}$ (1), $\text {C}_{{4}}\text {Cl}_{{2}}\text {F}_{{6}}$ (2)

If the molecular ion is absent, we give the detected maximal fragments instead. Note that several maximal fragments may be detected for one substance. The last column indicates if the correct molecular ion is reconstructed by our method, with its ranking in parenthesis, or which molecular ion(s) is reconstructed (if any - for brevity only the two first ranked wrong molecular ions are reported)

**Fig. 5:**
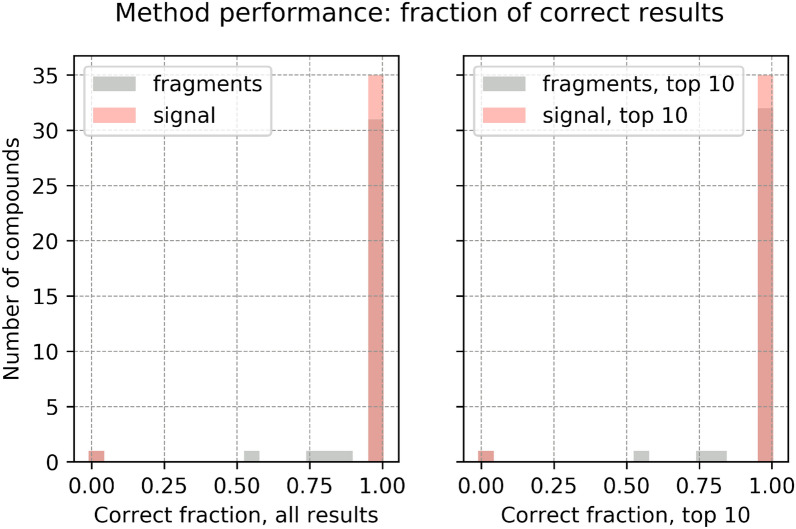
Performance of the identification algorithm: fraction of correct reconstructed fragments and signal. A fragment is considered correct if its chemical formula is a subset of the chemical formula of the true molecular ion. The histogram for fragments is shown in grey and for signal in peach. Left: fraction of correct reconstructed fragments compared to all reconstructed fragments (grey); fraction of correct reconstructed signal compared to the sum of reconstructed signal (peach). Right: fraction of correct fragments from the top-10 likelihood list of fragments (grey); fraction of the associated correct signal compared to the signal reconstructed by the top-10 likelihood fragments (peach). If the number of reconstructed fragments is not more than 10, then the top-10 results have same value as the results considering all fragments. Two substances have fragments poorly identified: $\text {CF}_{{4}}$ and $\text {SO}_{{2}}\text {F}_{{2}}$. See text for "Discussion"

**Fig. 6:**
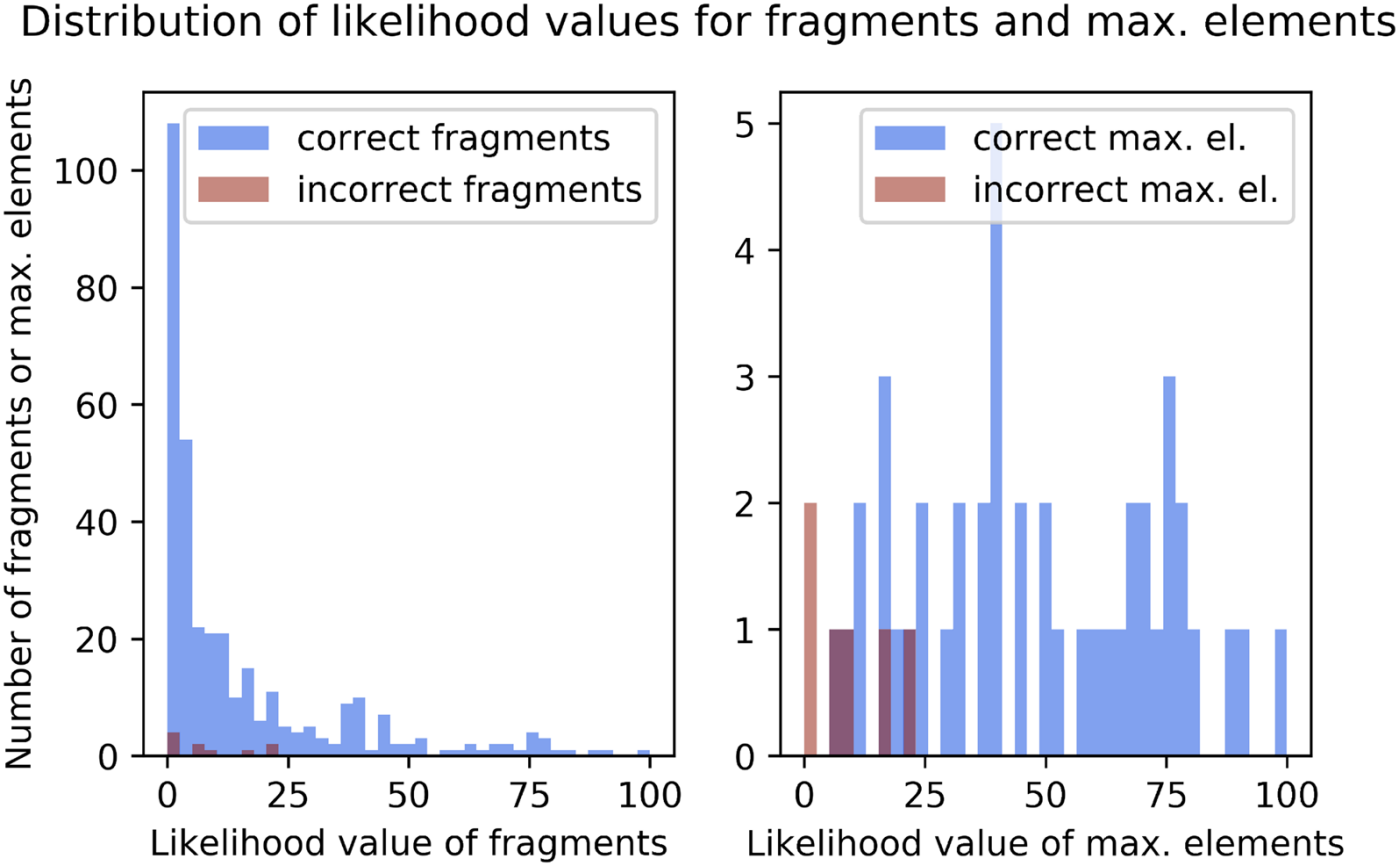
Training set: distribution of likelihood values of fragments (left) and maximal fragments (right). A likelihood value of 100 indicates that the chemical formula of the fragment or maximal fragment is highly likely. Blue: distribution for correctly identified fragments/maximal fragments. Red: distribution for wrongly identified fragments/maximal fragments. In total, there were 353 reconstructed fragments, 343 correct and 10 wrong, and 50 maximal fragments, 44 correct and 6 wrong. Above a likelihood value of 20, >95% of the fragments are correct, and >90% of the maximal fragments

Optimisation strategies to speed up the enumeration are reported in the Additional file 1 (Sect. 5.3). In particular, isotopocules with intensity expected below the instrumental limit of detection are not enumerated.

At the end of Step 3, knapsack-generated chemical formulae, containing only abundant isotopes, are organised as nodes in a pseudo-fragmentation graph. Each knapsack formula of a node is complemented by its minor-isotope formula(e) if the latter is above the instrumental limit of detection.

### Step 4: Computing the optimum contribution for each isotopocule set individually

We now consider the measured mass intensity profile. Potentially, any candidate chemical formula may contribute to the measured intensity profile along the mass axis. First, one generates the theoretical mass profile for each node, i.e. for each knapsack formula together with its minor-isotope formulae. Then, one optimises a certain contribution for each node taken individually, to match the measured mass profile.

#### Computing a profile of intensity vs mass for a given set of isotopocules

For each set of isotopocules belonging to the same node, we generate an expected mass profile. A measured intensity profile is not continuous, it is a discrete set of coordinates $(m_a, I_{m_a})$ where $m_a$ is a mass abscissa, and $I_{m_a}$ is the intensity for this mass. We consider that a knapsack fragment and its associated minor-isotope fragments have an expected intensity profile made of the sum of contributions of all isotopocules along the mass range. Each mass peak is generated as a pseudo-Voigt profile, with a prescribed peak width as obtained from the mass calibration (Additional file 1) and a mass resolution of about 8 ppm, which is sufficient given our instrumental mass resolution. We obtain an expected discrete mass profile (a set of coordinates) for the whole isotopocule set of the node, of the form $\{(m_a,{\tilde{I}}_{m_a}):m_a\in \text {mass abscissa}\}$.

#### Computing the contribution of a given set of isotopocules

At this point, over the mass domain covered by a given candidate set of isotopocules *i*, we look for a non-negative scaling factor $k_i$, such that the measured signal $s_{\text {measured}} = \{(m_a, I_{m_a}):m_a \in \text {mass abscissa}\}$ best fits the theoretical profile of the set. This can be seen as an optimisation problem, where the difference between measured and generated profile is minimised:

3 $$\begin{aligned} s_{\text {measured}} - k_i \cdot {\tilde{I}}_i = \sum _{m_a \in \text {mass abscissa}} \left( I_{m_a} - k_i \cdot {{\tilde{I}}}_{i,m_a}\right) = 0 \end{aligned}$$

where $s_{\text {measured}}$ and ${\tilde{I}}_i$ can be seen as vectors. With only one $k_i$ value to optimise, Eq. (3) can be simplified as the average value of the measured profile divided by the computed isotopocule profile:

4 $$\begin{aligned} k_i = \frac{1}{\#\{m_a \in \text {mass abscissa}\}} \sum _{m_a \in \text {mass abscissa}} \frac{I_{m_a}}{{{\tilde{I}}}_{i,m_a}} \end{aligned}$$

After optimisation of the factor $k_i$, if the entire profile falls below the LOD, the candidate node is removed from the graph of solutions.

This step can be seen as computing the maximum contribution for a given node (as if no other nodes were present to contribute to the signal). The computed maximum contribution, that we denote $k_{i}^{\max }$, will be used as starting value for the first computation of likelihood in Step 5.

### Step 5: Ranking of candidates according to a likelihood estimator

To help us select the most probably correct formulae among all candidate formulae, we define an artificial likelihood estimator based on two quantifications, as explained hereafter. The estimator takes as input a knapsack fragment, together with its set of minor-isotope fragments.

We decide to capture in the likelihood estimator the other knapsack fragments that are sub-fragments, and their isotopocules. For example, for $\text {CCl}_{{2}}$ as chosen knapsack formula, we would consider its five minor-isotope formulae (e.g. [$^{13}$C]$\text {Cl}_{{2}}$), the subformulae CCl and Cl and their own set of isotopocules (C of m/z 12 is filtered out in our TOF MS). At Step 4, we estimated $k_{i}^{\max }$, the maximum value that $k_i$ could take. We now estimate the maximum proportion *g* of signal that a candidate fragment *n*, its isotopocules, and all its sub-fragments *i* could explain. These considerations lead to a first possible estimator:

5 $$\begin{aligned} g(n) = \frac{\sum \limits _{i~\text {sub-fragment of}~n} k_i^{\max } \sum \limits _{j~\text {isotopocule of}~i}p_{i,j}}{\sum \limits _{m_{\text {spec}} \in \text {mass spectrum}} I_{m_{\text {spec}}}} \end{aligned}$$

where $p_{i,j}$ is the theoretical abundance of the given isotopocule *j* for the given fragment *i* computed with Eq. 7 in the Additional file 1, $m_{\text {spec}}$ ranges over the mass spectrum, $I_{m_{\text {spec}}}$ is the intensity at that mass ($I_{m_{\text {spec}}}$ is computed as a discrete integral, this is the area of the peak of the measured signal around $m_{\text {spec}}$). Numerical values of *g*(*n*) for knapsack fragments of $\text {CCl}_{{4}}$ are given in Table 5, fourth column. In practice, we have observed a misbehaviour of the estimator *g*(*n*) from Eq. (5): knapsack formulae that contain many atomic elements have many more sub-fragments, of various masses, hence a higher probability to match a larger fraction of the signal. Eq. (5) therefore gives advantage to “complicated” formulae. This effect can be seen for knapsack fragments of CFC-11 (Table 5, col. 4): fragment HCFCl (wrong) gets a higher score than CFCl (correct) using Eq. (5). Misbehaviour of similar estimators, also capturing the total matched signal, has been reported previously [62].

To correct for this effect, we multiply Eq. (5) by the number of found sub-formulae, divided by the expected maximum number of sub-formulae. In practice, this maximum number of sub-formulae is computed with the knapsack algorithm, using the minimum detectable mass as lower bound (in our case, $m/z = 23$, as all smaller masses are filtered out in our TOF detector). As the number of knapsack solutions increases with i) the total number of atoms and ii) the number of elements present in the fragment, using this number as denominator will favour solutions constituted of a limited number of elements. The advantage of this technique is to favour “simple” solutions, without setting any parameter to limit the number of elements to use. Equation (5) is therefore completed as:

6 $$g(n) = \frac{\sum\limits_{i\text{ sub-fragment of }n} k_i^{\max} \sum\limits_{j \text{ isotopocule of } i}p_{i,j}} {\sum\limits_{m_{\text{spec}} \in \text{mass spectrum}} I_{m_{\text{spec}}}} \frac{\# \{\text{sub-fragments }i\text{ of }n\}} {\# \{ \text{all theoretical sub-fragments of }n\text{, mass }\geq 23m/z \}}$$

where $\# \{\text {sub-fragments}~i~\text {of}~n\}$ is the number of existing subfragments in the directed graph, for fragment *n*.

All knapsack fragments are set out in order by decreasing likelihood value. Looking again at the same example for CFC-11 in Table 5, using Eq. (6) now fragment CFCl (correct) gets a higher likelihood score than HCFCl (col. 5), and is therefore ranked better (col. 6). We underline that this defined likelihood estimator has no chemical signification. Its aim is only to highlight the simplest knapsack solutions that explain the maximum proportion of the measured signal. It is therefore only a practical tool to speed up the identification process.

**Table 5:** Behaviour of the likelihood estimator: ten first knapsack fragments for CFC-11 and $\text {CCl}_{{4}}$, set out in order according to their likelihood value calculated at the first iteration of Step 5. Some fragments may be deleted at subsequent iterations

Fragment	Exact mass	% Assigned signal of the fragment	% Assigned signal of all (sub)fragments	Likelihood	Ranking	Maximal fragment?
CFC-11 ($\text {CFCl}_{{3}}$)						
$\text {CFCl}_{{3}}^{+}$	135.90496	0.0	98.4	88.6	1	True
$\text {CFCl}_{{2}}^{+}$	100.93611	81.9	96.3	84.3	2	False
CFCl$^{+}$	65.96726	5.6	11.9	11.9	3	False
*CHFCl$^{+}$	66.97508	0.0	12.0	9.3	4	True
$\text {CCl}_{{3}}^{+}$	116.90656	2.1	10.0	8.0	5	False
* $\text {CHCl}_{{3}}^{+}$	117.91438	0.0	10.2	7.9	6	True
$\text {CCl}_{{2}}^{+}$	81.93771	2.6	8.0	6.0	7	False
* $\text {CHCl}_{{2}}^{+}$	82.94553	0.0	8.1	5.8	8	False
CCl$^{+}$	46.96885	3.1	5.4	5.4	9	False
$\text {C}_{{3}}\text {Cl}_{{2}}^{+}$	105.93771	0.0	8.0	3.2	10	False
$$\text {CCl}_{{4}}$$						
$\text {CCl}_{{3}}^{+}$	116.90656	71.2	92.4	73.9	1	False
* $\text {CHCl}_{{3}}^{+}$	117.91438	0.4	93.9	73.0	2	True
* $\text {CHCl}_{{2}}^{+}$	82.94553	0.5	22.3	16.0	3	False
$\text {CCl}_{{2}}^{+}$	81.93771	11.6	21.2	15.9	4	False
*$\text {COCl}_{{2}}^{+}$	97.93262	0.3	21.8	12.1	5	True
CCl$^{+}$	46.96885	5.5	9.5	9.5	6	False
* HCl$^{+}$	35.97668	0.7	4.7	4.7	7	False
Cl$^{+}$	34.96885	4.0	4.0	4.0	8	False
* $\text {CF}_{{2}}$Cl$^{+}$	84.96566	0.2	9.7	3.6	9	True
* $\text {OCl}_{{2}}^{+}$	85.93262	0.3	4.3	2.2	10	False

$1^{\mathrm{st}}$ column: chemical formula of the fragment, containing only abundant isotopes. * indicates that the fragment is not part of the molecular ion. $2^{\mathrm{nd}}$ col.: calculated exact mass of ionised fragment. $3^{\mathrm{rd}}$ col.: percent signal of the fragment and its isotopocules relative to the total measured signal. $4^{\mathrm{th}}$ col.: percent signal of the fragment, all its sub-fragments and all associated isotopocules relative to the total measured signal, computed from Eq. (5). $5^{\mathrm{th}}$ col.: likelihood value computed from Eq. (6). $6^{\mathrm{th}}$ col.: ranking according to decreasing likelihood value. For CFC-11, $\text {CFCl}_{{3}}$ ranked first is the molecular ion

### Step 5 to Step 8: Iterating loop to compute the optimum contribution of multiple sets of candidates together

Overall, the measured signal profile $s_{\text {measured}} = \{(m_a, I_{m_a}):m_a \in \text {mass abscissa}\}$ should match a linear combination of the expected profiles of all correct candidate sets, an approach already found in e.g. [63]. Formally, this approach allows several fragments to form together the signal of one measured peak, which is realistic given our mass resolution. Equation (3) is therefore completed as:

7 $$\begin{aligned} s_{\text {measured}} - \sum k_i \cdot {\tilde{I}}_i = \sum _{m_a \in \text {mass abscissa}} \left( I_{m_a} - \sum _{\text {profile}~i} k_i \cdot {{\tilde{I}}}_{i,m_a}\right) = 0 \end{aligned}$$

This equation cannot be simplified. Instead, the optimisation of multiple $k_i$ is handled by a machine learning technique, using the Python package lmfit, based on the Levenberg-Marquardt algorithm [for details, see 64]. This optimisation of multiple $k_i$ together is by far the most expensive computation step in the entire method. The lmfit package is most efficient when only a limited number of parameters $k_i$ are optimised. In particular, it is outside the computing power of a regular desktop machine to optimize all the profiles of the candidate formulae together. We implement three techniques to lower the running time of lmfit.

First, the measured profile is itself not perfect but affected by measurement noise. This noise also affects the expected isotopic pattern accuracy. To account for this, we do not aim at reconstructing 100% of the signal, but only a significant portion of it. Since our experimental precision is in the order of 1 % to 5%, as well as the isotopic pattern accuracy, we set the threshold at 95% of the signal: when 95% of the “area” below the signal is reconstructed, the optimisation is stopped. The fraction of explained signal *f* is calculated as follow, iterating over the *i* selected candidate fragments, their respective set of isotopocules, and the mass spectrum:

8 $$\begin{aligned} f = \frac{ \sum \limits _{\text {selected candidate}~i}~ k_i \sum \limits _{\text {isotopocule}~j~\text {of}~i}p_{i,j}}{\sum \limits _{m_{\text {spec}} \in \text {mass spectrum}}I_{m_{\text {spec}}}} \end{aligned}$$

where $k_i$ are the linear factors optimised by the lmfit package. We aim at reaching $f \ge 0.95$.

Second, a further reduction of computation time is achieved by splitting the mass domain: all candidates are grouped into smaller, non-overlapping mass domains, where the optimisation is run separately. Indeed, optimising a small number of $k_i$ multiple times is more efficient than optimising a large number of $k_i$ just at once.

Third, we observe that at this stage, many wrong solutions generated by the knapsack are still present, while usually, only a limited number of chemical formulae are really present (see later discussion in "How wrong knapsack solutions are rejected and implications for computation runtime" section  and also Fig. 8). We therefore adopt the following greedy-like strategy^5^, in order to reduce the number of fitted isotopic profiles. The most likely solutions are processed first, until the reconstructed signal reaches 95% of the measured signal. This way, unlikely solutions left after 95% of the signal has been reconstructed are not considered at all. This approach requires all candidate fragments to be ordered according to a well chosen likelihood estimator, as done in Step 5.

In practice, from the list of ranked knapsack formulae (or nodes on the graph), we take the one ranked first, together with all its sub-fragments (or children of the node) and all associated isotopocules, optimise the contributions of these selected nodes (Step 7), and then eliminate any node below the LOD, as well as any node becoming a singleton (Step 8). Since the $k_i$ have updated values, the ranking is updated (back to Step 5). If less than 95% of the signal is reconstructed, the next most likely node is added to the selected nodes (Step 6). This iterating procedure is depicted in Fig. 1, green boxes.

**Fig. 7:**
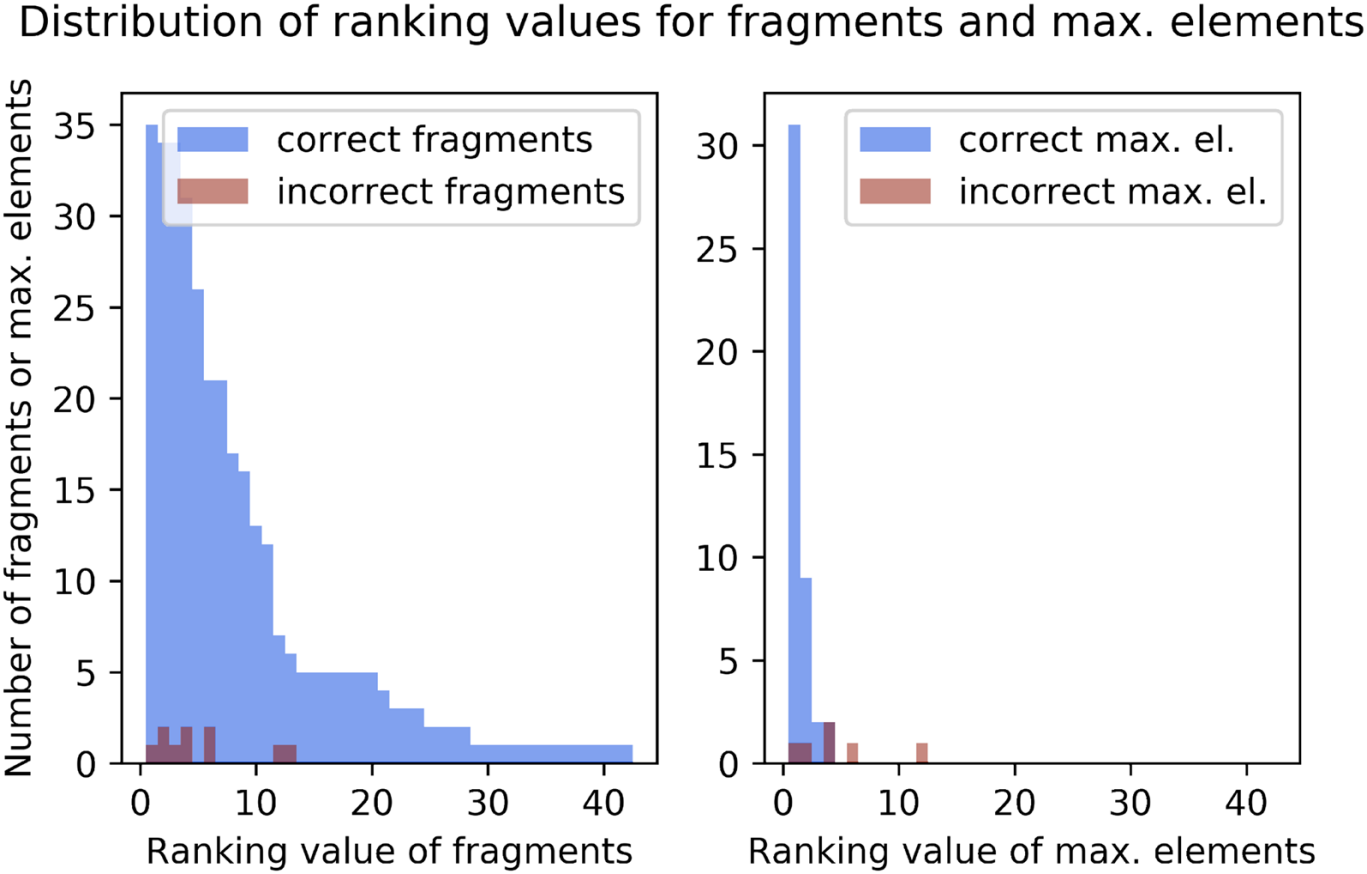
Training set: distribution of ranking values for fragments and maximal fragments. A ranking value of 1 means that the fragment/maximal fragment was ranked as most likely (maximum likelihood value within the set of fragments/maximal fragment). Blue: distribution of ranking for correctly identified fragments/maximal fragments. Red: distribution of ranking for wrongly identified fragments/maximal fragments

**Fig. 8:**
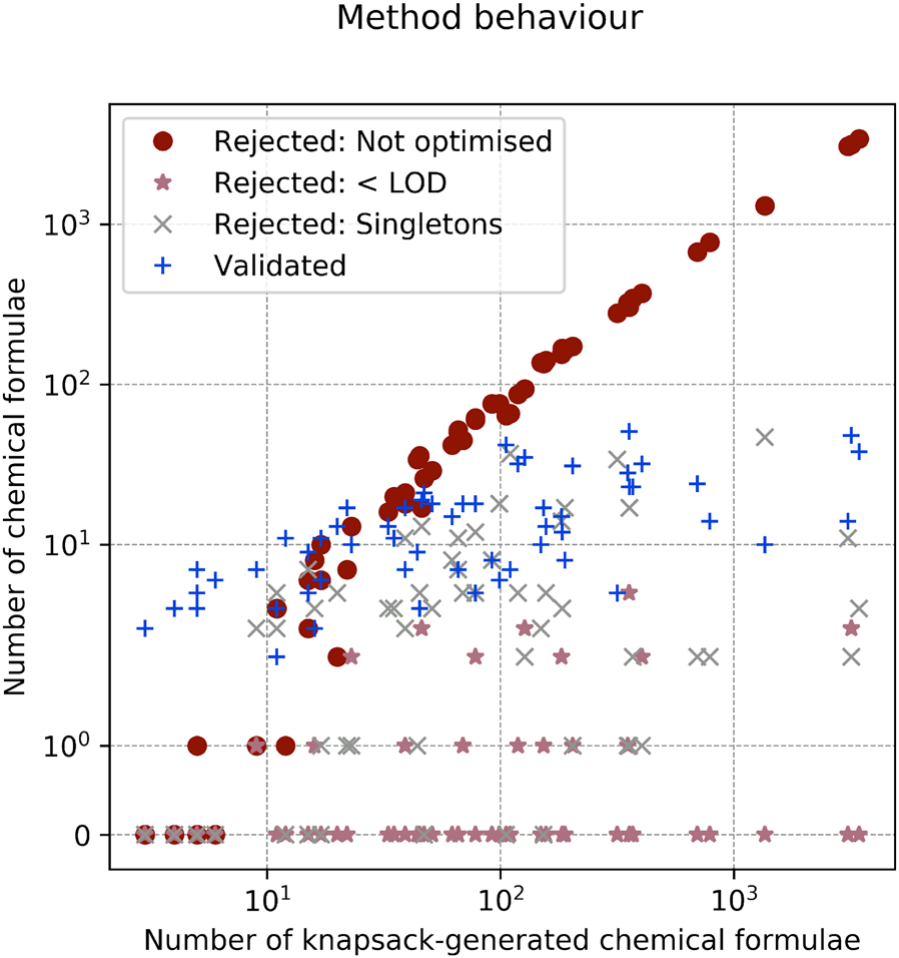
Behaviour of the identification algorithm for the training and validation sets: how knapsack solutions are rejected

At the end of this iterating procedure, nodes that have not been selected to be optimised are deleted from the pseudo-fragmentation graph, and any new singletons are deleted as well (Step 9). For each remaining node, its factor $k_i$ is used to compute the final contribution of this node to each measured mass peak.

### Step 10: Tentative identification of the molecular ion

At the end of Step 9, the majority of detected fragments have been assigned a chemical formula. Using this information, we develop a simple algorithm to identify or reconstruct likely molecular ions. The three conditions of the SENIOR theorem have to be fulfilled, as listed by Kind and Fiehn [57]:

1. The sum of valences or the total number of atoms having odd valences is even.
2. The sum of valences is greater than or equal to twice the maximum valence. This rule prevents fragments such as CFCl to be considered as valid molecular ion.
3. The sum of valences is greater than or equal to twice the number of atoms minus 1. In our settings, by construction all fragment formulae have a non-negative DBE value, therefore this rule is fulfilled.

We start from the list of maximal fragments still part of the graph at the end of Step 9, and separate them into two groups, with odd or even sum valence.

All maximal fragments with even valence fulfil the first condition of the SENIOR theorem. We then test for the second condition. All maximal fragments fulfilling these two criteria are added to the list of potential molecular ions.

Using all the maximal fragments with odd valence, we enumerate all possibilities of adding one monovalent atom to each of them, using all monovalent atoms present in all fragments on the graph. Each newly constructed maximal fragment, if fulfilling the second SENIOR condition, is considered a potential molecular ion. It is added to the graph with all other fragments, and its likelihood value is computed.

This algorithm implicitly makes the assumption that all multivalent atoms present in the true molecule have been detected in at least one fragment and correctly identified.

## Results and discussion

### Validation data from standard measurements

To evaluate the reconstructed fragment formulae, we adopt the following pragmatic approach, already used in e.g. [65]: a fragment formula is considered to be correct if it is a sub-formula of the true molecular formula. Fragments resulting from re-arrangements, which do happen with EI ionisation, are thus considered as correct solutions.

### Estimators of method performance

A first quantitative measurement of method performance is how much of the measured signal $I_{m_{\text {spec}}}$ is reconstructed, written $f_{I\!,{{\,\mathrm{total}\,}}}$, irrespective of whether the signal corresponds to correct or incorrect fragments:

9 $$\begin{aligned} f_{I\!,{{\,\mathrm{total}\,}}} = \frac{ \sum I_{{{\,\mathrm{correct~fragment}\,}}} + I_{{{\,\mathrm{incorrect~fragment}\,}}}}{\sum \limits _{m_{\text {spec}} \in \text {mass spectrum}} I_{m_{\text {spec}}}} \end{aligned}$$

Reconstruction of fragment formulae produces a qualitative information. To quantify method performance, we construct two metrics based on recommendations for qualitative measurements [66], Annex D].

We define the ratio of correctly identified fragment formulae $f_n$ as the number of correctly identified fragment formulae divided by the total number of reconstructed fragment formulae, per compound:

10 $$\begin{aligned} f_{n,{{\,\mathrm{correct}\,}}} = \frac{n_{{{\,\mathrm{correct~fragment}\,}}}}{n_{{{\,\mathrm{correct~fragment}\,}}} + n_{{{\,\mathrm{incorrect~fragment}\,}}}} \end{aligned}$$

Then, we define the ratio of correctly assigned signal $f_{I\!,{{\,\mathrm{correct}\,}}}$ as the sum of intensities of correctly assigned fragments divided by the total intensity of all reconstructed fragments:

11 $$\begin{aligned} f_{I\!,{{\,\mathrm{correct}\,}}} = \frac{\sum I_{{{\,\mathrm{correct~fragment}\,}}}}{ \sum I_{{{\,\mathrm{correct~fragment}\,}}} + I_{{{\,\mathrm{incorrect~fragment}\,}}}} \end{aligned}$$

For comparison, we also compute per training compound the fraction of correct fragments and signal on the top-10 results, which are the list of maximum 10 fragments with maximum likelihood.

### Performances of the method on the training set

An example of the output produced by the identification method is plotted in Fig. 4. All numerical values can be found in the Additional file 3 (see section Additional Information). What is delivered to the user is a list of the chemical formulae of the generated and non-eliminated fragments, their exact mass, assigned signal, likelihood value according to Eq. (6) and ranking. The method also informs if a fragment is a maximal fragment and if it is a potential molecular ion fulfilling the SENIOR theorem. For example, for CFC-11 the maximal fragment $\text {CFCl}_{{3}}$ with ranking of 1 (maximum likelihood) is the molecular ion (Table 5). This example demonstrates the usefulness of our likelihood estimation to identify the fragments closest to, or being, the molecular ion.

**Table 6:** Numerical values for the obtained runtime on the training set, in seconds

Compound	No. knapsack solutions	Step 1: Knapsack	Step 2: Graph	Step 3: Iso. Enum.	Step 7: Optimisation	Total
$\text {C}_{{2}}\text {H}_{{6}}$	4	0	0	0.00035	0.58114	0.61831
$\text {C}_{{3}}\text {H}_{{8}}$	12	0.00100	0.00103	0.00277	0.93511	1.02196
$\text {CH}_{{3}}$Cl	6	0.00100	0	0.00100	0.22414	0.28026
COS	5	0.00175	0	0.00100	0.05579	0.09874
$\text {NF}_{{3}}$	11	0.00100	0	0.00089	0.09805	0.11600
Benzene	47	0.00399	0.00798	0.01695	1.36925	1.54867
$\text {CH}_{{2}}\text {Cl}_{{2}}$	15	0.00739	0.00100	0.00499	0.29121	0.39700
HCFC-22	17	0.00390	0.00100	0.00320	0.23608	0.31227
$\text {CF}_{{4}}$	3	0.00068	0	0.00100	0.04567	0.06479
Toluene	106	0.00894	0.03889	0.05063	1.23265	1.67224
$\text {CH}_{{3}}$Br	15	0.00484	0.00100	0.00345	0.06066	0.09972
HCFC-142b	127	0.00697	0.03890	0.03164	3.55514	3.90335
$\text {SO}_{{2}}\text {F}_{{2}}$	16	0.00299	0.00099	0.00399	0.19949	0.23456
CFC-13	9	0.00298	0	0.00099	1.99773	2.02956
HCFC-141b	46	0.00499	0.00603	0.01056	1.52992	1.65464
$\text {CHCl}_{{3}}$	17	0.00939	0.00105	0.00499	0.08471	0.16614
CFC-12	35	0.01390	0.00794	0.01178	0.28523	0.42641
$\text {C}_{{2}}\text {HCl}_{{3}}$	66	0.01396	0.01615	0.02093	0.09776	0.30321
CFC-11	44	0.01503	0.00757	0.01496	0.14162	0.30119
HCFC-124	110	0.01198	0.02792	0.02887	0.10436	0.34243
PFC-116	78	0.00891	0.01794	0.02439	0.08703	0.34109
$\text {CH}_{{3}}$I	45	0.01097	0.00499	0.01476	1.05608	1.17037
$\text {SF}_{{6}}$	99	0.00901	0.02990	0.03683	0.05829	0.35405
Halon-1301	39	0.01396	0.00895	0.01894	0.12531	0.26130
$\text {CCl}_{{4}}$	23	0.01103	0.00200	0.00470	0.78681	0.86528
CFC-115	183	0.01696	0.11013	0.07779	0.42488	1.15757
$\text {C}_{{2}}\text {Cl}_{{4}}$	92	0.06682	0.02865	0.05261	0.09374	0.54953
Halon-1211	78	0.01994	0.01795	0.02094	0.50948	0.73878
CFC-114	358	0.02792	0.36901	0.17453	0.47408	2.15301
$\text {CH}_{{2}}\text {Br}_{{2}}$	11	0.02094	0.00047	0.00233	0.06566	0.11770
CFC-113	698	0.06004	1.08788	0.42879	0.61436	4.44405
PFC-218	317	0.01794	0.27526	0.13997	0.06965	2.87218
$\text {SF}_{{5}}\text {CF}_{{3}}$	66	0.00698	0.00997	0.01695	0.08213	0.23013
PFC-c318	149	0.01241	0.04537	0.06582	0.09975	0.58743
Halon-2402	1362	0.12367	4.24480	0.78490	0.14161	11.59514
$\text {C}_{{6}}\text {F}_{{14}}$	3096	0.09473	23.70908	1.79919	0.21742	76.37336

**Fig. 9:**
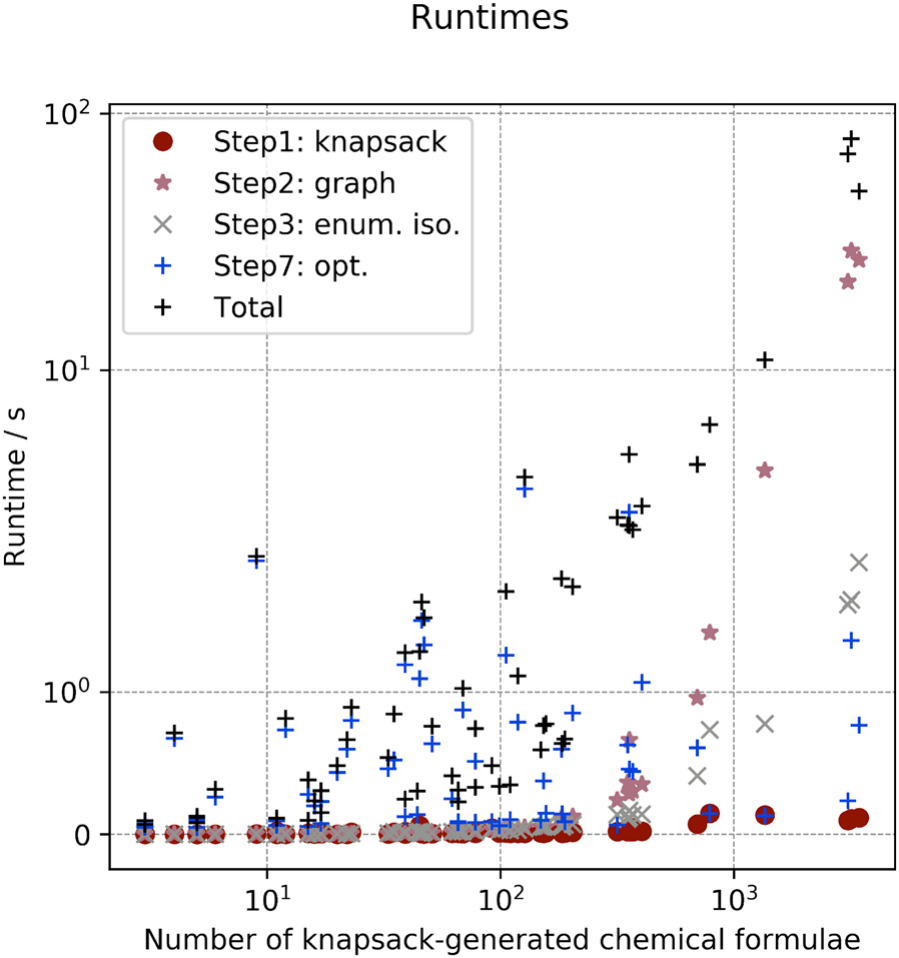
Runtimes of the identification algorithm for the training and validation sets. The total runtime per compound is shown in black. The runtime of specific steps is also depicted: for the knapsack (Step 1), for the graph construction with all knapsack fragments (Step 2), for the enumeration of all minor-isotope chemical formulae above the LOD (Step 3), for the optimisation of contribution of sets of fragments, using a machine learning algorithm (Step 7). For most compounds, Step 7 remains the most time intensive step. The corresponding numerical values are given in Tables 6 and 7

These values are displayed on Fig. 9. Step 1: knapsack enumeration of fragment formulae. Step 2: graph construction. Step 3: isotopocule enumeration. Step 7: optimisation of multiple isotopocule sets together using lmfit

**Fig. 10:**
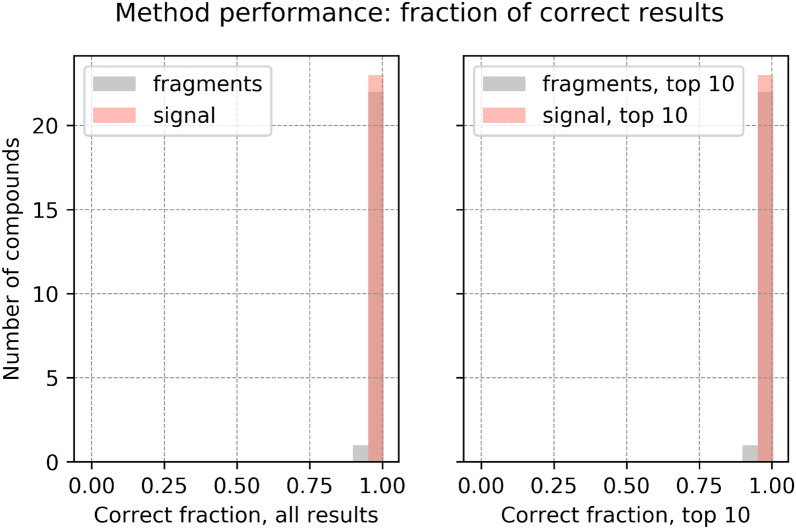
Performance of the identification algorithm on the validation set (21 compounds): fraction of correct reconstructed fragments and signal. A fragment is considered correct if its chemical formula is a subset of the chemical formula of the true molecular ion. The histogram for fragments is shown in grey and for signal in peach. Left: fraction of correct reconstructed fragments compared to all reconstructed fragments (grey); fraction of correct reconstructed signal compared to the sum of reconstructed signal (peach). Right: fraction of correct fragments from the top-10 likelihood list of fragments (grey); fraction of the associated correct signal compared to the signal reconstructed by the top-10 likelihood fragments (peach). If the number of reconstructed fragments is not more than 10, then the top-10 results have same value as the results considering all fragments

#### Fraction of reconstructed signal

For 34 compounds in the training set (all but two), the fraction of reconstructed signal is above 0.95. For $\text {CF}_{{4}}$ and $\text {C}_{{2}}\text {H}_{{6}}$, it is 0.06 and 0.88, respectively. In these two cases, the measured mass and its uncertainty envelope did not contain the true mass, causing the knapsack to fail in generating the correct chemical formulae. Given that we multiply the mass uncertainty with a coverage factor of 2.5 (Eq. (1)), corresponding to 98.5% of the expected mass interval, it can be expected that in a few cases, the considered mass domain does not contain the true answer. We observe that no wrong fragment fills this gap, but the signal is rather not reconstituted. It is therefore easier for the user to identify such extreme cases, and e.g. run the identification process again using a larger coverage factor.

For $\text {CF}_{{4}}$, correct chemical formulae were reconstructed for the three measured masses when using a coverage factor of 6.0. For $\text {C}_{{2}}\text {H}_{{6}}$, using $k=2.5$, no solution is produced for the measured fragment at 29.037805 m/z. Using a coverage factor *k* of 3.0 instead allows the knapsack to produce the correct fragment formula, $\text {C}_{{2}}\text {H}_{{5}}$.

#### Compounds with very few measured masses

As a consequence of their simple molecule structure or a very low abundance in the sample, five compounds had fewer than 6 detectable masses ($\text {NF}_{{3}}$, $\text {CF}_{{4}}$, $\text {CH}_{{3}}$I, $\text {SO}_{{2}}\text {F}_{{2}}$, $\text {SF}_{{5}}\text {CF}_{{3}}$). We observed that in such cases, our identification algorithm does not have enough constrains to suggest correct results. This confirms previous observations from Hufsky et al. [21]. We therefore developed the following strategy: when the number of measured masses is less than 6, maximal fragments are treated separately through the iterative Step 5 to Step 8 (green boxes on Fig. 1), so that chemical formulae belonging to different maximal fragments are not optimised together. The list of kept maximal fragments is then returned as result, ordered by likelihood. The program returns a message warning that multiple maximal fragments are possible, and suggests the user to choose the one considered most likely.

For the sake of including these compounds with all other results in the following figures, the maximal fragment ranked first is kept, and all others are eliminated (assuming no co-elution). In all cases except for $\text {SO}_{{2}}\text {F}_{{2}}$, where only two masses were measured, the first ranked maximal fragment was correct.

#### Fraction of correct reconstructed fragments and signal

Fig. 5 displays the histograms of the fractions of correct fragment formulae and of correct reconstructed signal, for all fragments (left) and for the top-10 fragments for each compound (right). Fig. 5 shows better fraction of correct results when taking the fraction of correct signal into account compared to the fraction of correct chemical formulae: wrongly identified fragments tend to have a smaller abundance, mostly due to higher mass uncertainty or lack of companion peak that would provide an isotopic constrain. For only 44% of the compounds, 90% or more of the chemical formulae are correct. However, for more than 90% of the compounds, the signal from correct fragments constitutes at least 90% of the reconstructed signal. This underlines that the proportion of signal assigned to each chemical formula carries information about how likely this chemical formula is correct.

The ability of the method to produce correct chemical formulae further improves when taking into account the top-10 results only, i.e. the 10 chemical formulae ranked as most likely according to the likelihood estimation, and their associated signal. For these top-10 fragments, the chemical formulae are at least 90% correct for 58% of the compounds; the proportion of compounds for which at least 90% of the signal is correct stays unchanged, at 90%. Indeed, most of the time, wrong fragments are anyway assigned a small portion of the signal.

For the training set, only two compounds were poorly identified, $\text {CF}_{{4}}$ and $\text {SO}_{{2}}\text {F}_{{2}}$, for reasons discussed previously.

#### Information from the likelihood estimation and ranking results

As we have seen, the identification algorithm does not eliminate all wrong chemical formulae. We now study more in detail the likelihood value and ranking associated to each reconstructed fragment to see if these values can better inform us if a fragment is correct or wrong.

Fig. 6 (left histogram) presents the distribution of likelihood values for correct fragments (in blue) and for incorrect fragments (in red), and the same for maximal fragments only (right histogram). From these distributions, we observe that likelihood values above 20 indicate that the fragment is correct by 95% (*n* = 89, 85 correct and 4 incorrect fragments), and the maximal fragment correct by 90% (*n* = 35, 32 correct and 3 incorrect maximal fragments). We could therefore use a likelihood value threshold above which a fragment or maximal fragment could be tagged as most probably correct. At the other end of the distribution, 90% of maximal fragments with a likelihood value below 8 are wrong (*n* = 31, 3 correct and 28 incorrect maximal fragments). In contrast, small likelihood values do not necessarily indicate that the fragment is false, but rather that the fragment represents a small portion of the signal. Only below a likelihood value of 0.3, 90% of the fragments are wrong. This implies that using a given likelihood value as cut off to delete fragments would be either inefficient and delete very few fragments, or be inaccurate and delete many correct fragments.

The distribution of ranking values for fragments and maximal fragments are shown in Fig. 7. The left histogram illustrates that the ranking of fragments does not help separating between correct and wrong fragments because the corresponding distributions overlap strongly. However, for the maximal fragments, this overlap is less pronounced (right histogram): correct maximal fragments have a ranking value usually better than 10, while maximal fragments whose ranking is worse than this value are mostly wrong.

**Fig. 11:**
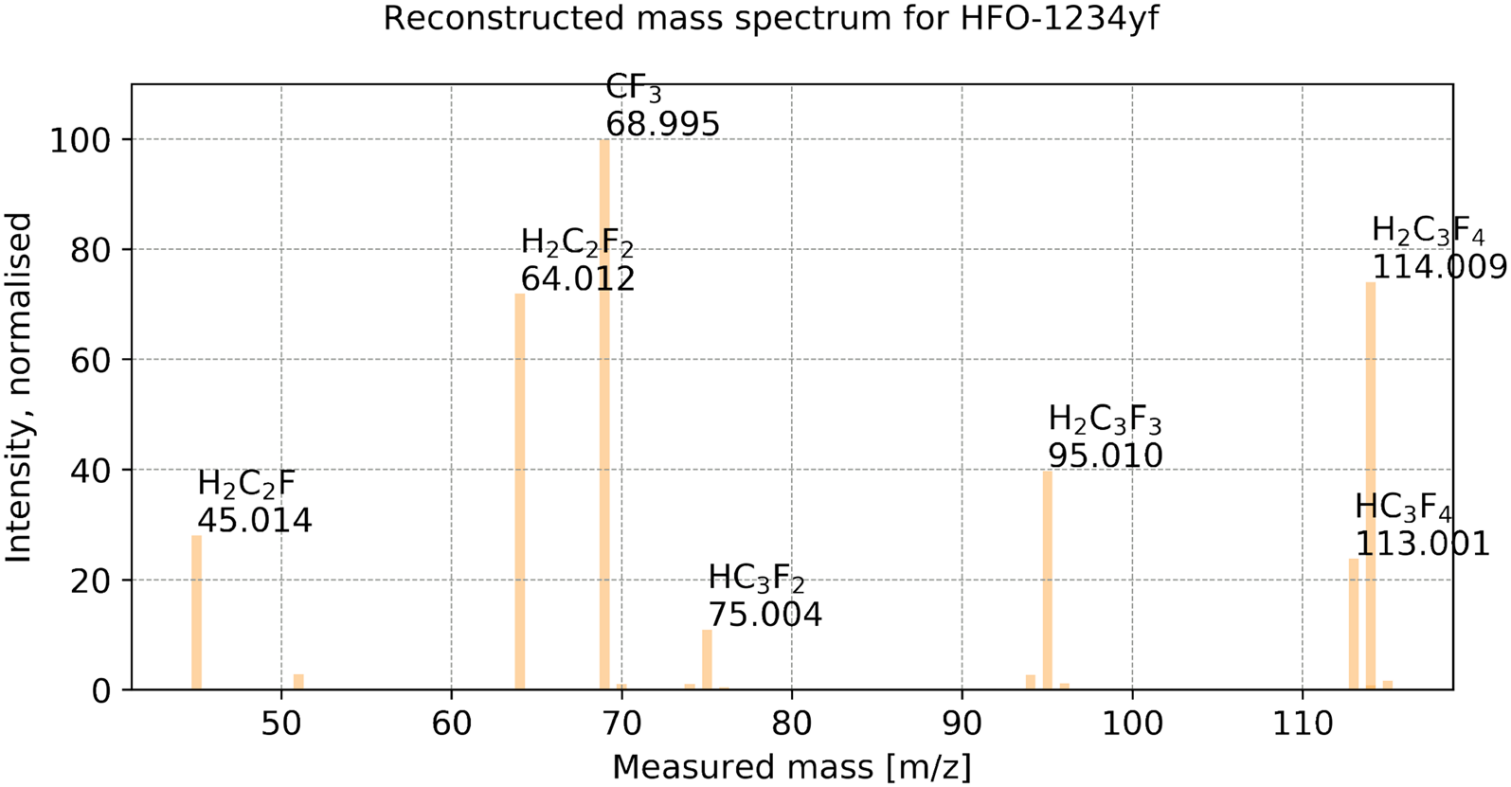
Reconstructed mass spectrum for HFO-1234yf, when setting as target that 95% of the signal should be reconstructed. Numerical values can be found in the Additional file 3

These observations can help the user in the identification process by tagging the maximal fragments with a likelihood value above 20 or a ranking value better than 10 as probably correct, and the others as likely wrong.

#### Reconstructed molecular ions

For 29 molecules on the training set (>80 %), the reconstructed molecular ion ranked first is the correct one (see detailed results in Tables 3). For $\text {CCl}_{{4}}$ and HCFC-141b, the correct solution is ranked second and the solution ranked first is still quite close to the correct solution ($\text {CHCl}_{{3}}$ instead of $\text {CCl}_{{4}}$ and $\text {C}_{{2}}\text {H}_{{4}}$FCl instead of $\text {C}_{{2}}\text {H}_{{3}}\text {FCl}_{{2}}$, respectively). For PFC-c318, perfluorohexane and $\text {SF}_{{5}}\text {CF}_{{3}}$, sub-fragments of the molecular ion are listed. These last three cases are wrong because our reconstruction method assumes that the correct number of multivalent atoms is detected in at least one fragment, which was not the case. For $\text {CF}_{{4}}$ and $\text {SO}_{{2}}\text {F}_{{2}}$, no correct solution was suggested, these two cases are discussed in "Fraction of reconstructed signal" and "Compounds with very few measured masses" sections.

For comparison, we tested two software that suggest the weight of the molecular ion. Both software use unit mass information, not high resolution. We used the same training set, excluding $\text {C}_6\text {F}_{{14}}$ because its molecular weight is outside our mass detection range. The NIST library tool [22] was able to reconstruct the correct molecular weight in 17 cases ($\approx$49 %). In average, the NIST molecular weight prediction deviates from the correct one by 12 Da, with a median deviation of 2 Da, which is very similar to the performances published initially in 1993 by Scott et al. [22]. We also tested the commercial software MOLGEN-MS, which contains a module to determine if specific chemical classes are present in a molecule (MSclass module, [25]), and then predicts the weight of the molecular ion (ElCoCo module, [23]). We found better results when using the ElCoCo module alone, without MSclass: the correct weight for the molecular ion was listed in 15 cases ($\approx$43 %). Technical details are given in the Additional file 1.

### Performance of the model on the validation set

The validation set (Table 2) is made of 23 compounds. The performance of the reconstructions is similar as with the training set: for 95% of the compounds, more than 90% of the reconstructed signal is correct (Fig. 10). This illustrates that our identification method can be applied to different dataset, while producing similar performances. As an example, the reconstructed mass spectrum for HFO-1234yf is given in Figure 11.

**Table 7:** Numerical values for the obtained runtime on the validation set, in seconds

Compound	No. knapsack solutions	Step 1: Knapsack	Step 2: Graph	Step 3: Iso. Enum.	Step 7: Optimisation	Total
HFC-41	3	0.00098	0	0.00199	0.0748	0.09376
HFC-32	5	0.001	0	0.001	0.14962	0.17655
HFC-152	20	0.00199	0.00198	0.00499	0.48074	0.53358
HFC-152a	22	0.00199	0.00199	0.00598	0.63579	0.70561
HFC-23	5	0.00099	0	0.00299	0.10472	0.13266
HFC-143	33	0.00299	0.00299	0.00698	0.56412	0.64393
HFC-143a	39	0.00499	0.00798	0.00898	1.28358	1.38034
HFC-134	51	0.00499	0.00997	0.01396	0.75513	0.8778
HFC-134a	69	0.00598	0.01396	0.01795	1.15894	1.34445
HFC-125	62	0.00899	0.01297	0.01795	0.31715	0.51916
HFC-245ca	119	0.00897	0.04189	0.05086	1.04231	1.44332
HFC-245fa	204	0.01695	0.10971	0.06982	0.73306	1.49405
HFC-365mfc	356	0.02094	0.40094	0.13765	3.05672	5.10083
HFC-236cb	404	0.02693	0.43587	0.16956	0.91956	3.0124
HFC-236ea	352	0.01995	0.35804	0.13165	0.58447	2.39165
HFC-236fa	157	0.01297	0.06383	0.06148	0.17143	0.85424
HFC-227ea	369	0.02592	0.34808	0.15559	0.49173	2.80308
HFC-43-10mee	3192	0.1516	27.8139	1.71247	1.455	83.46999
HFO-1234yf	184	0.00798	0.08976	0.06084	0.15259	0.72908
HFO-1234ze(E)	153	0.00997	0.04887	0.04488	0.44783	0.87914
HCFO-1233zd(E)	189	0.01296	0.10572	0.07579	0.08976	0.74725
HCBD	790	0.13464	1.53374	0.77896	0.14859	6.21535
TCHFB	3452	0.15757	28.7482	2.01913	0.85859	52.94723

These values are displayed on Fig. 9. Step 1: knapsack enumeration of fragment formulae. Step 2: graph construction. Step 3: isotopocule enumeration. Step 7: optimisation of multiple isotopocule sets together using lmfit

The reconstructed molecular ion is correct in 19 cases (83 %, see detailed results in Table 4). For the wrong cases, the suggested molecular ions are mostly sub-fragments of the true molecular ion. Interestingly, when the correct molecular ion is listed, it is always ranked first, suggesting that the likelihood estimator (Eq. 6) is quite effective in ranking first the most likely results.

### How wrong knapsack solutions are rejected and implications for computation runtime

Figure 8 displays, for each compound of the training and validation sets, how many knapsack fragments are generated, kept and rejected. For compounds where less than $\approx$50 knapsack fragments were generated, we observe that most fragments have gone through the iterating steps of the workflow (see Fig. 1), and are therefore validated (Fig. 8, blue crosses) or deleted, as singletons (grey ’x’) or as being below the LOD (mauve stars). On the other hand, for compounds with more than $\approx$100 knapsack fragments, the number of fragments gone through the computation-intensive iterating steps do not exceed $\approx$100, even if more than 1000 knapsack fragments were generated. In such cases, most fragments are rejected at Step 9 of the workflow (Fig. 1). This behaviour may explain why the computation runtime does not increase linearly with the number of generated knapsack fragments, as discussed hereafter.

Details about the computation runtime are given in Fig. 9. For all compounds, the knapsack step (Step 1) represents only a minor part of the runtime, proving that the optimisation of this computation step is appropriate for the considered compounds. Above 1000 generated knapsack formulae, the graph construction (Step 2) represents an important portion of the runtime, but remains minor for all compounds with less than 1000 knapsack fragments. Since the runtime for the graph construction is proportional to the number of generated knapsack solutions, if applied to larger molecules, further optimisation will be necessary to limit the computation time. For example, a method to pre-select likely present chemical elements may be useful.

For most compounds, the most computation intensive step is the optimisation of multiple isotopocule profiles (Step 7), which uses the machine learning tool lmfit. However, the runtime of Step 7 does not increase linearly with the number of knapsack solutions, but for most compounds is limited to less than 10 seconds. We attribute this to the fact that only a limited number of most likely knapsack fragments go through this expensive step.

## Conclusion

Adequate information about the presence in the environment and potentially illicit emissions of halogenated and in particular (per)fluorinated compounds is more and more pressing, requiring a broader use of NTS approaches [67]. Gas chromatography followed by electron ionisation and high-resolution mass spectrometry is increasingly used for ambient air quality measurements and is a promising technique for non-target screening of atmospheric trace gases. To support automated identification of small unknown (halogenated) substances, especially in cases where the molecular ion is absent from the obtained mass spectrum, specific data analysis tools making use of newly availably high resolution mass spectrometry data are expected to improve current identification performances.

In this work, we have developed a novel algorithm to allow reconstruction of the chemical formula based on the measured mass of fragments likely belonging to the same substance. The developed method specifically addresses the observation that peaks with low signal have a higher mass uncertainty, by using a specific uncertainty for each measured mass peak. This approach allows to use all measured data simultaneously, while giving more weight to more accurate mass peaks. Our method does not require the molecular ion to be present, and can still reconstruct the chemical formula of all other detected fragments. This is important for electron-ionisation spectra, where the molecular ion is absent in approx. 40% of the cases. In addition, we developed a simple algorithm to generate and rank possible molecular ions, based on the addition of likely monovalent chemical elements to the largest identified fragments.

Overall, the method performs well on very heterogeneous compounds, comprising nine different chemical elements and many different structures, for molecules from 3 atoms (COS) to 20 atoms ($\text {C}_6\text {F}_{{14}}$), over a large molar mass domain from 30 g $\text {mol}^{-1}$ to over 300 g $\text {mol}^{-1}$, and measured with an average mass uncertainty of 70 ppm: for more than 90% of the compounds, more than 90% of the signal has been assigned to the correct chemical formula. The presented method was able to reconstruct and rank first the molecular ion in >80 % of the cases. The reconstruction becomes less reliable with decreasing number of detected mass peaks, in case of compounds measured at very low molar fraction.

Finally, we would like to emphasis that in the difficult field of compound annotation and structure elucidation, robust knowledge can certainly be gained from applying different methods in parallel. For example, when the retention time information is available, additional confidence could be gained from comparison with predictions (e.g., using Quantitative Structure Property Relationships (QSPR) models or correlations with the boiling point, as previously done in e.g. [35]). For the compounds where the molecular ion was proven present in the spectrum, the fragmentation tree computation method from Hufsky et al. [21] could then be applied. For further structure elucidation, the identified or generated most likely molecular ion could be fed to subsequent molecular structure generators and EI fragmentation programs, which already exists [14, 29, 31–33, 68, 69].

## Supplementary Information

**Additional file 1**: Main supplementary information.

**Additional file 2**. Zip file containing all input mass spectral data for ALPINAC, for the training and validation sets. There is one text file per compound.

**Additional file 3**. Zip file containing all output formulae produced by ALPINAC, for the training and validation sets, using the default settings. There is one text file per compound.

**Additional file 4**. Zip file containing all input mass spectral data, converted to .TRA files compatible with MOLGEN-MS.

**Additional file 5**. ZIP file containing all input mass spectral data, converted to .jdx files compatible with the NIST EI library.

**Additional file 6**. Latex version of the supplementary material (Additional file 1), format .tex.

## Data Availability

The Python code is released with the acronym ALPINAC, for ALgorithmic Process for Identification of Non-targeted Atmospheric Compounds, under an open-source license at https://gitlab.inria.fr/guillevi/alpinac/. In the future we will also release it at https://gitlab.empa.ch. In addition, the final published version will be archived on the Zenodo repository. All measured mass spectra are provided in a zip Additional file 2, in a .txt format compatible with ALPINAC. The same data are also provided as .jdx (NIST compatible, see Additional file 5) and .tra (MOLGEN-MS compatible, see Additional file 4) formats, in zip files.

## Footnotes

1 H, $^{2}$H, C, $^{13}$C, N, $^{15}$N, O, $^{17}$O, $^{18}$O, F, S, $^{33}$S, $^{34}$S, Cl, $^{36}$S, $^{37}$Cl, Br, $^{81}$Br, I.

2 B, Si, P and noble gases are also supported by ALPINAC and can be added to the list of chemical elements to use if needed.

3 A variant of the knapsack problem was used to build cryptosystems to securely hide secrets in the 70’s. It was later broken with the LLL algorithm. We leave to future work the application of the LLL algorithm to our setting.

4 in the usual mathematical meaning, e.g. https://en.wikipedia.org/wiki/Partially_ordered_set#Extrema.

5 https://en.wikipedia.org/wiki/Greedy_algorithm.
