# Supplementary material for: Automated fragment formula annotation for electron ionisation, high resolution mass spectrometry: application to atmospheric measurements of halocarbons
Source: J Cheminform. 2021 Oct 4;13:78. doi: 10.1186/s13321-021-00544-w (PMC8491408; doi:10.1186/s13321-021-00544-w)
Supplement: Supplementary file 1 — Additional file 1: Main supplementary information. [file 13321_2021_544_MOESM1_ESM.pdf]

SUPPLEMENT

Supplement for: Automated fragment formula annotation for electron ionisation, high resolution mass spectrometry: application to atmospheric measurements of halocarbons

Myriam Guillevic[1*], Aurore Guillevic[2], Martin K. Vollmer[1], Paul Schlauri[1], Matthias Hill[1], Lukas Emmenegger[1] and Stefan Reimann[1]

[*]Correspondence:
myriam.guillevic@empa.ch
[1]Laboratory for Air Pollution /Environmental Technology, Empa, Swiss Federal Laboratories for Materials Science and Technology, Ueberlandstrasse 129, 8600 Dübendorf, Switzerland
Full list of author information is available at the end of the article

1 Training set and validation set: SMILES codes

We provide here the SMILES codes for each of the substances in the training and validation set and if a mass spectrum is documented in the NIST spectral database [1].

Table 1 Known compounds used as training set: CAS numbers, SMILES codes and presence in the NIST chemistry webbook [1]. All values are taken from the ChemSpider website [2], the PubChem website [3] and the NIST website [1].

Compound	Chemical formula	CAS number	SMILES code	NIST spectrum
C_2H_6	C_2H_6	74-84-0	CC	yes
C_3H_8	C_3H_8	74-98-6	CCC	yes
CH_3Cl	CH_3Cl	74-87-3	CCl	yes
COS	COS	463-58-1	C(=O)=S	yes
NF_3	NF_3	7783-54-2	N(F)(F)F	yes
Benzene	C_6H_6	71-43-2	C1=CC=CC=C1	yes
CH_2Cl_2	CH_2Cl_2	75-09-2	C(Cl)Cl	yes
HCFC-22	HCF_2Cl	75-45-6	C(F)(F)Cl	yes
CF_4	CF_4	75-73-0	C(F)(F)(F)F	yes
Toluene	C_7H_8	108-88-3	CC1=CC=CC=C1	yes
CH_3Br	CH_3Br	74-83-9	CBr	yes
HCFC-142b	$H_3C_2F_2Cl$	75-68-3	CC(F)(F)Cl	yes
SO_2F_2	SO_2F_2	2699-79-8	O=S(=O)(F)F	yes
CFC-13	CF_3Cl	75-72-9	C(F)(F)(F)Cl	yes
HCFC-141b	$H_3C_2FCl_2$	1717-00-6	CC(F)(Cl)Cl	yes
$CHCl_3$	$CHCl_3$	67-66-3	C(Cl)(Cl)Cl	yes
CFC-12	CF_2Cl_2	75-71-8	C(F)(F)(Cl)Cl	yes
C_2HCl_3	C_2HCl_3	79-01-6	C(=C(Cl)Cl)Cl	yes
CFC-11	$CFCl_3$	75-69-4	C(F)(Cl)(Cl)Cl	yes
HCFC-124	HC_2F_4Cl	2837-89-0	C(C(F)(F)F)(F)Cl	yes
PFC-116	C_2F_6	76-16-4	C(C(F)(F)F)(F)(F)F	yes
CH_3I	CH_3I	74-88-4	CI	yes
SF_6	SF_6	2551-62-4	FS(F)(F)(F)(F)F	no
Halon-1301	CF_3Br	75-63-8	C(F)(F)(F)Br	no
CCl_4	CCl_4	56-23-5	C(Cl)(Cl)(Cl)Cl	yes
CFC-115	C_2F_5Cl	76-15-3	C(C(F)(F)Cl)(F)(F)F	yes
C_2Cl_4	C_2Cl_4	127-18-4	C(=C(Cl)Cl)(Cl)Cl	yes
Halon-1211	CF_2ClBr	353-59-3	C(F)(F)(Cl)Br	yes
CFC-114	$C_2F_4Cl_2$	76-14-2	C(C(F)(F)Cl)(F)(F)Cl	yes
CH_2Br_2	CH_2Br_2	74-95-3	C(Br)Br	yes
CFC-113	$C_2F_3Cl_3$	76-13-1	C(C(F)(Cl)Cl)(F)(F)Cl	yes
PFC-218	C_3F_8	76-19-7	C(C(F)(F)F)(C(F)(F)F)(F)F	yes
SF_5CF_3	SF_5CF_3	373-80-8	C(F)(F)(F)S(F)(F)(F)(F)F	yes
PFC-c318	C_4F_8	115-25-3	C1(C(C(C1(F)F)(F)F)(F)F)(F)F	yes
Halon-2402	$C_2F_4Br_2$	124-73-2	C(C(F)(F)Br)(F)(F)Br	yes
C_6F_{14}	C_6F_{14}	355-42-0	C(C(C(C(F)(F)F)(F)F)(F)F)(C(C(F)(F)F)(F)F)(F)F	yes

Table 2 Known compounds used as validation set: CAS numbers, SMILES codes and presence in the NIST chemistry webbook [1]. All values are taken from the ChemSpider website [2], the PubChem website [3] and the NIST website [1].

Compound	Chemical formula	CAS number	SMILES code	NIST spectrum
		Kigali Amendment to the Montreal Protocol		
HFC-41	H_3CF	593-53-3	CF	yes
HFC-32	H_2CF_2	75-10-5	C(F)F	yes
HFC-152	$H_4C_2F_2$	624-72-6	C(CF)F	yes
HFC-152a	$H_4C_2F_2$	75-37-6	CC(F)F	yes
HFC-23	HCF_3	75-46-7	C(F)(F)F	yes
HFC-143	$H_3C_2F_3$	430-66-0	C(C(F)F)F	yes
HFC-143a	$H_3C_2F_3$	420-46-2	CC(F)(F)F	yes
HFC-134	$H_2C_2F_4$	359-35-3	C(C(F)F)(F)F	yes
HFC-134a	$H_2C_2F_4$	811-97-2	C(C(F)(F)F)F	yes
HFC-125	HC_2F_5	354-33-6	C(C(F)(F)F)(F)F	yes
HFC-245ca	$H_3C_3F_5$	679-86-7	C(C(C(F)F)(F)F)F	yes
HFC-245fa	$H_3C_3F_5$	460-73-1	C(C(F)F)C(F)(F)F	no
HFC-365mfc	$H_5C_4F_5$	406-58-6	CC(CC(F)(F)F)(F)F	no
HFC-236cb	$H_2C_3F_6$	677-56-5	C(C(C(F)(F)F)(F)F)F	no
HFC-236ea	$H_2C_3F_6$	431-63-0	C(C(F)F)(C(F)(F)F)F	yes
HFC-236fa	$H_2C_3F_6$	690-39-1	C(C(F)(F)F)C(F)(F)F	yes
HFC-227ea	HC_3F_7	431-89-0	C(C(F)(F)F)(C(F)(F)F)F	no
HFC-43-10mee	$H_2C_5F_{10}$	138495-42-8	C(C(C(F)(F)F)F)(C(C(F)(F)F)(F)F)F	no
		HFOs		
HFO-1234yf	$H_2C_3F_4$	754-12-1	C=C(C(F)(F)F)F	no
HFO-1234ze(E)	$H_2C_3F_4$	29118-24-9	C(=CF)C(F)(F)F	no
HCFO-1233zd(E)	$H_2C_3F_3Cl$	102687-65-0	C(=CCl)C(F)(F)F	no
		Halogenated compounds with high boiling point		
HCBD	C_4Cl_6	87-68-3	C(=C(Cl)Cl)(C(=C(Cl)Cl)Cl)Cl	yes
TCHFB	$C_4Cl_4F_6$	375-45-1	C(C(C(F)(F)Cl)(F)Cl)(C(F)(F)Cl)(F)Cl	no

2 Mass calibration procedure

A time-of-flight (ToF) instrument measures a time, elapsed between two events: the extraction and the detection, when the ions hit the detector plate. To convert this time measurement into a mass measurement in the most possible accurate manner, internal mass calibration proves to be a good strategy. Known masses detected during a measurement are used to establish the calibration function between ToF and mass. In our cases, we use known masses produced by fragmentation of a mass calibration substance, perfluoroperhydrophenanthrene (PFPHP), of chemical formula $C_{14}F_{24}$. Only two types of atoms are present in this molecule, carbon (mass 12.000000) and fluorine (mass 18.99840316). The most abundant peaks can therefore be associated to a unique molecular formula. For example, the peak at integer mass 69 can be associated to CF_3^+ only, of exact mass 68.99466108.

Based on the NIST mass spectrum and our measured mass spectrum for PFPHP, we have chosen a list of 13 masses present with a sufficient abundance to be used for mass calibration. In addition, we use the masses of N_2, O_2, Ar, which are the most abundant air components and may slightly leak in our system, as well as Cl, also observed to be always present in our detector. The masses are chosen to be evenly distributed between the minimum and maximum masses, covering a range from m/z = 28.0055994348 (N_2^+) to m/z = 292.98188618 ($C_7F_{11}^+$).

We have observed that our ToF detector is subject to mass drift of up to 100 ppm during a run. This drift is possibly due to temperature variation of our preceding GC. To correct for this drift, we perform a mass calibration every two minutes, using average data of the preceding and following two minutes. For each set of four-minutes-averaged data, all peaks in the mass domain near the exact masses of the selected list are detected. Note that several mass peaks can be detected where only one exact mass from the calibrant is expected. Each detected peak is fitted using a pseudo-Voigt function, which is a combination of a Gaussian and a Lorentzian function:

$$f(x; A, \mu, \sigma, \alpha, b) = (1-\alpha)\frac{A}{\sigma\sqrt{2\pi}} \exp\left(-\frac{(x-\mu)^2}{2\sigma^2}\right) + \alpha\frac{A}{\pi}\left(\frac{\sigma_L}{(x-\mu)^2 + {\sigma_L}^2}\right) + b \quad (1)$$

where

$$\sigma_L = \sigma\sqrt{2\ln(2)} \quad (2)$$

and the full-with-at-half-maximum (FWHM)

$$\mathrm{FWHM} = 2\sigma\sqrt{2\ln(2)} = 2\sigma_L \; . \quad (3)$$

The parameter FWHM is used later on (Main article, Section 2.5.1) to generate candidate mass peaks with the appropriate peak broadness.

Then, all obtained centres of ToF are associated to the closed expected exact mass. The entire set of pairs (ToF; expected exact mass) is used to fit a calibration function of the form:

$$i_{\mathrm{ToF}} = p_1 m^{p_3} + p_2 \quad (4)$$

with i_{ToF} the time of flight index and m the exact mass. The parameters p_1, p_2 and p_3 are optimised using the Python lmfit package. According to theory, p_3 should be equal to 0.5 [1]. However a better accuracy is obtained when p_3 is allowed to slightly vary. If one or several pairs are further away from the fit than a set maximum value (20 ppm in our case), the furthest away pair is eliminated and the optimisation routine is repeated. This one-by-one pair elimination improves the robustness of the algorithm. This was proven necessary as when measuring real air from the industrial area where Empa is located, once in while a prominent pollution event occurs, producing outstanding mass peaks that may be in the vicinity of the expected peak, even masking it, therefore disturbing the mass calibration function.

Once all residuals are below the set value, the mass calibration is complete. Any ToF value can then be converted to a m/z value using:

$$m = \left(\frac{i_{\mathrm{ToF}} - p_2}{p_1}\right)^{\frac{1}{p_3}} \quad (5)$$

where p_1, p_2 and p_3 are calculated at any given specific time as linear interpolation using their time-bracketing optimised values.

3 Uncertainty of the mass calibration

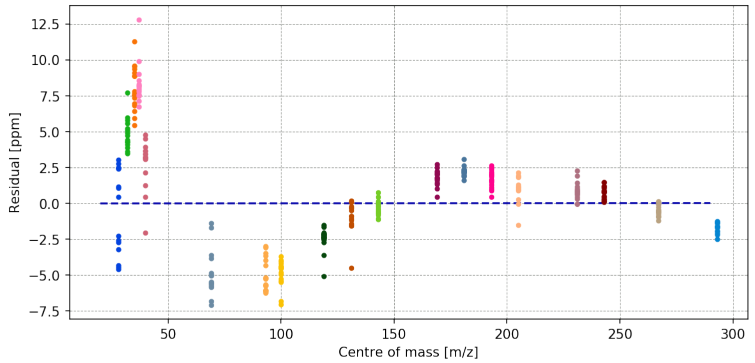

Figure 1 Residuals of the mass calibration vs mass. Dots of the same colour represent the same mass peak, but at a different time slices.

After the optimisation, the obtained fit parameters are used to calculate the reconstructed m/z values; these values are then compared to the expected exact m/z, for each time slice of four minutes. The obtained offsets, expressed in ppm over the mass domain, are displayed in Fig. 1. With our instrument, the observed mass accuracy is better for larger masses, with residuals usually below 5 ppm for masses higher than 100 m/z, while the accuracy can deteriorate to 20 ppm below 50 m/z. This is potentially due to the mass resolution of our instrument that is around 3000 for masses smaller than 50 m/z but around 4000 for larger masses.

To reflect this varying mass accuracy over the mass domain, for each used exact mass, we use as uncertainty the maximum observed offset at this mass. Then for any measured mass, its uncertainty is calculated as a linear interpolation between uncertainty at bracketing masses. This constitutes the mass calibration uncertainty.

[1] https://en.wikipedia.org/wiki/Time-of-flight_mass_spectrometry

4 Validation set: preparation of qualitative standards for compounds newly regulated by the Kigali amendment to the Montreal Protocol

Eighteen substances listed under the Kigali Amendment to the Montreal Protocol were part of the validation set.

First, substances were separated in two groups, with the aim that each group should not contain isomers, to make sure each substance could be identified by its mass spectrum only. Group A contained: HFC-41, HFC-143a, HFC-134a, HFC-227ea, HFC-236ea, HFC-245fa, HFC-43-10-mee, HFC-152 and HFC-236cb. Group B contained: HFC-32, HFC-23, HFC-125, HFC-152a, HFC-365mfc, HFC-143, HFC-236fa, HFC-245ca and HFC-134.

The pure substances were bought from Synquest Laboratories (Florida, USA). For each group, the pure substances were spiked one after the other into synthetic air, and the mixture was pressurised into a flask. The two obtained mixtures were prepared at approximately 6.5 nmol·mol^{-1}.

Then, each mixture was measured by our preconcentration, gas chromatography, time-of-flight mass spectrometry instrumentation. Data analysis then followed the same procedure as explained in the main article.

5 Algorithmic improvements

We describe our algorithmic improvements to speed-up the running-time of some critical steps.

5.1 Organisation of the chemical formulae in a directed acyclic graph (DAG)

After running the knapsack algorithm, many candidate chemical formulae are obtained, hereafter simply referred to as 'formulae'. We organise them in a graph. In this graph, a node n_j is a descendant of a node n_i if the node's fragment s_j is a sub-fragment of the fragment s_i of the node n_i. For example, CCl is a descendant of CCl_3. Conversely, a node n_i is an ancestor of a node n_j if its fragment s_i is a sup-fragment of the fragment s_j. This define a *partial order* on the formulae that we formally define in Definition 1.

Definition 1 (Partial order) We define the following partial order on the formulae. Let s_i and s_j be two formulae encoded as vectors of non-negative integers.

- The formula s_j is *smaller than* the formula s_i, denoted $s_j \leq s_i$, if s_j is a sub-fragment of s_i;
- The formula s_j is *greater than* the formula s_i, denoted $s_j \geq s_i$, if s_j is a sup-fragment of s_i;
- otherwise s_i and s_j are *incomparable.*

Organising a set $\mathcal{S}$ of n items (here fragments) in a DAG (directed acyclic graph) can takes as many as n^2 comparisons of items, but since $\mathcal{S}$ can be quite large (e.g., $n = 10000$), it makes sense to reduce the number of comparisons. Moreover, the graph should have as less edges as possible, that is, two formulae $s_i \geq s_j$ are binded with an edge if and only if there is no other formula $s_{i'}$ that could be inserted between them like $s_i \geq s_{i'} \geq s_j$. For example with CCl, CCl_2 and CCl_3, we will define the graph with minimal edges on the left, not the one of the right (Fig. 2).

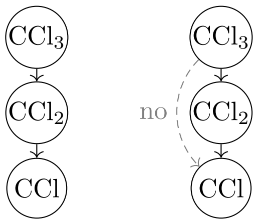

Figure 2 Directed acyclic graph.

We now explain how we reduced the number of comparisons between fragments to set the edges, thus improving the complexity of building the graph. First the target masses are sorted in decreasing order before the knapsack step. Hence the output of the knapsack is made of batches of chemical formulae, each one for a given target mass, in decreasing order. *Because the mass uncertainty is far thinner than* $0.5m/z$*, all chemical formulae for one target mass are* incomparable*: it is not possible to find that a formula is a sub-fragment of another, for the same target mass, otherwise the mass difference between the two would be at least* $1m/z$*.*

Therefore we have a list of formulae $\{s_i\}_{1\leq i\leq \#\mathcal{S}}$ such that for any formula s_i, the formulae in the preceeding batches weight more and are either incomparable or contain s_i as a sub-fragment, and the formulae in the forthcoming batches are lighter and either incomparable or sub-fragments of s_i. The maximal fragments will be the nodes at the "roots" of the DAG. They are made of the formulae of the first (heaviest) batch, and some other formulae from other batches.

We maintain a list of the root nodes (maximal fragments) of the graph. They have no ancestor, and they are incomparable to each other. To add a new formula in the graph, because of the ordering of the formulae, we know that it is either incomparable, or a subfragment of any node of the graph. *It cannot be a sup-fragment (a parent) of any node of the graph.* We compare the new formula to each of the maximal fragments. If it is incomparable to any of them, we add it as as new maximal fragment. Otherwise, for each maximal fragment that has the new formula as sub-fragment, we compare the new one to its children. If it is incomparable to any of the children, we add it as a new child of the maximal fragment (we add an edge toward it). Otherwise, we recursively explore the children of the children that have this new formula as sub-fragment. In this way, we avoid many useless comparisons: all children of incomparable nodes are omitted.

Thanks to the list of maximal fragments, the singletons are identified right away: they are the maximal fragments without any child. Figure 2n the main article shows the graph obtained for CCl_4.

5.2 Removing a node and updating the edges

A node n to be removed has parents (closest sup-fragments) connected with one edge, and children (closest sub-fragments) connected with one edge. We wrote this procedure to remove a node and update the edges and list of maximal fragments (Alg. 1, example in Fig. 3).

Algorithm 1: remove the node n and update the edges

for *each parent p_i of n* **do**
  remove the edge from p_i to n ;
  for *each child c_i of n* **do**
    lists its own parents q_j (without n) ;
    if *none of q_j is a sub-fragment of p_i* **then**
      add an edge from p_i to c_i (if there is one q_j which is a sub-fragment of p_i, do nothing: the edges are already fine)
for *each child c_i of n* **do**
  remove the edge from n to c_i ;
  if *c_i has no more parent after removing n* **then**
    add it in the list of maximal fragments
if *n is a maximal fragment (it has no parent)* **then**
  remove it from the list of maximal fragments
Remove n

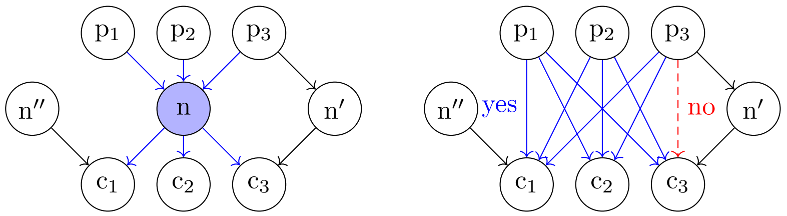

Figure 3 Example for Algorithm 1: removing a node and updating the edges.

5.3 Enumeration of isotopocules

We now recall the computation of the relative intensities of the minor isotopocules (see [4] for a detailed computation). The abundant formula has proportion (of the set of all isotopocules) $pr = \prod_{\{\text{element } e\}} (a_{e,0})^{n_e}$. The product is over all the distinct atoms (denoted e), $a_{e,0}$ is the abundance of the most abundant isotope of an atom, and n_e is the number of occurrences of that atom in the chemical formula. For example, with CCl_4 one computes $pr = a_C a_{Cl}^4 = 0.326$. An isotopocule with only one element e and i minor isotopes of abundance $a_{e,i}$ has proportion

$$\begin{aligned} pr_e &= a_{e,0}^{n_{e,0}} \binom{n_e}{n_{e,0}} a_{e,1}^{n_{e,1}} \binom{n_e - n_{e,0}}{n_{e,1}} a_{e,2}^{n_{e,2}} \binom{n_e - n_{e,0} - n_{e,1}}{n_{e,2}} \cdots a_{e,i}^{n_{e,i}} \binom{n_e - n_{e,0} - n_{e,1} - \ldots - n_{e,i-1}}{n_{e,i}} \\ &= a_{e,0}^{n_{e,0}} a_{e,1}^{n_{e,1}} a_{e,2}^{n_{e,2}} \cdots a_{e,i}^{n_{e,i}} \frac{n_e!}{n_{e,0}! n_{e,1}! n_{e,2}! \cdots n_{e,i}!} \end{aligned}$$

where the terms $\binom{n}{m} = \frac{n!}{m!(n-m)!}$ are binomal coefficients with $n \geq m$, denoting the number of ways to choose m items in a set of size n. Their product simplifies in $n_e!/(n_{e,0}!n_{e,1}!n_{e,2}! \cdots n_{e,i}!)$. An isotopocule has proportion

$$pr = \prod_e n_e! \prod_i (a_{e,i})^{n_{e,i}} / (n_{e,i}!) \tag{6}$$

where e ranges over the elements, i ranges over the isotopes of an element, n_e is the total number of an element (with all isotopes), $n_{e,i}$ is the number of occurences of one isotope. The relative intensity of a minor-isotope formula is the ratio (see [4])

$$p = \frac{\prod_e n_e! \prod_i (a_{e,i})^{n_{e,i}} / (n_{e,i}!)}{\prod_e (a_{e,0})^{n_e}} = \prod_e n_e! \prod_{i>0} \left(\frac{a_{e,i}}{a_{e,0}} \right)^{n_{e,i}} \frac{1}{n_{e,i}!} \; . \tag{7}$$

One needs to enumerate all the possible combinations of isotopes of a given element. This is a classical problem in combinatorics. Denote by i the number of isotopes (the abundant one included). Enumerate all the ways to sum at most i positive integers to obtain n_e. It can be seen as a knapsack-like problem: given all isotopes each of "weight" 1, finds how to sum to n_e. In particular, each isotope is allowed at most n_e times.

All ratios are relative to the abundant chemical formula, whose maximum possible intensity is known: this is the intensity of the corresponding measured mass. To improve the running-time of the enumeration, we do not list the minor isotopocules whose intensity would be below the detection threshold of the ToF-MS. For this purpose, we consider the elements one after the other. We maintain a list of partial isotopocules with their partial relative intensity, made of the elements processed so far. The list is ordered by decreasing relative intensity. Given a new element e and its occurence n_e, we generate its isotopic patterns, the abundant one included, and sort them in decreasing order of relative intensity. Reading the two lists in decreasing order of relative intensity, we combine the new isotopes to the partial solutions and multiply together the intensities. A loop over a list stops as soon as the product of intensities is below the partial threshold. Once the new list of partial solutions is computed, it is sorted in decreasing order of relative intensity. Then, the next element is processed in the same manner, until all elements are done. The first item of the resulting list is the isotopocule of highest relative intensity (it can be greater than one). For implementation purpose, we scale the list and divide all numbers by this highest relative intensity, so that everything is in the interval $[0, 1]$.

6 Full Numerical Example with carbon tetrachloride

For CCl_4 found at a retention time of 1708.27 s, nineteen masses are observed, listed in Table 7 with uncertainty and intensity.

6.1 Knapsack algorithm with two lists but without considering separately multi-valent and mono-valent atoms

In this paragraph we present an example of a knapsack algorithm with two sets of atoms, and two lists of intermediate masses. The candidate atoms are H, C, N, O, F, S, Cl, Br, I. We arbitrarily define two subsets: {C, N, O, S, Br} and {H, F, Cl, I}. The knapsack algorithm is run with input the masses of the atoms of each set, and the minimal mass is set to 1. One obtains two lists: A and B given in Table 5. The lists are sorted in increasing order of mass. One obtains A = [(12.0, C), (14.0030740074, N), (15.9949146223, O), (24.0, C_2), (26.003074007400002, CN), (27.9949146223, CO), (28.0061480148, N_2), (29.9979886297, NO), (31.97207073, S), (31.9898292446, O_2)] and B = [(1.0078250319, H), (2.0156500638, H_2), ...(all $H_{3...17}$), (18.140850574199998, H_{18}), (18.99840316, F), (19.1486756061, H_{19}), (20.0062281919, HF), ...(all $H_{20...33}$ and $H_{2...14}F$), (34.1157786385, $H_{15}F$), (34.2660510846, H_{34}), (34.96885271, Cl)]. Then pairing the masses of the two lists, one obtains masses within the target interval (if any): there is one solution (34.96885271, Cl). Finally, the DBE is computed and solutions with a negative DBE are discared. This first approach has a major drawback: many impossible sub-fragments are enumerated, im particular because of the Hydrogen which as a very small mass, and is mono-valent.

Here is a detailed example to compute the lists A and B for the first target mass $m_{\mathrm{max}} = 34.97006625322406$. First one computes multiples of each mass up to m_{max}. One obtains Tables 3 and 4. Then one combines at most one mass per column, starting the enumeration with the lightest mass of each column (the first row value). One obtains the fragments of the first row of Table 5.

Table 3 Initial partial list of masses before computing the list A made of fragments of atoms {C, N, O, S, Br} and masses up to $m_{\mathrm{max}} = 34.97006625322406$.

#	C	N	O	S	Br
1	12	14.0030740074	15.9949146223	31.97207073	
2	24	28.0061480148	31.9898292446		

Table 4 Initial partial list of masses before computing the list B made of fragments of atoms {H, F, Cl, I} and masses up to $m_{\mathrm{max}} = 34.97006625322406$. There are 34 multiples of Hydrogen: H, H_2, up to H_{34}.

	H	F	Cl	I
1	1.0078250319	18.99840316	34.96885271	
2	2.0156500638			
⋮	⋮			
34	34.2660510846			

Table 5 Intermediate lists in the knapsack algorithm with two sets of atoms {C, N, O, S, Br} for the list A and {H, F, Cl, I} for the list B. The notation $H_{1\text{-}34}$ means all the 34 fragments made of one to 34 atoms of Hydrogen. The notation $C_{1\text{-}2}O$ means CO and C_2O.

target mass interval	#A	A	#B	B	all sol.		DBE ≥ 0	
34.96751070677594, 34.97006625322406	10	S, $O_{1\text{-}2}$, NO, CO, $N_{1\text{-}2}$, CN, $C_{1\text{-}2}$	51	Cl, $H_{1\text{-}15}F$, F, $H_{1\text{-}34}$	1	Cl	1	Cl
35.974137795964566, 35.97778836403543	10	S, $O_{1\text{-}2}$, NO, CO, $N_{1\text{-}2}$, CN, $C_{1\text{-}2}$	54	HCl, Cl, $H_{1\text{-}16}F$, F, $H_{1\text{-}35}$	1	HCl	1	HCl
36.96406557296814, 36.967505987031856	11	S, $O_{1\text{-}2}$, NO, CO, $N_{1\text{-}2}$, CN, $C_{1\text{-}3}$	56	HCl, Cl, $H_{1\text{-}17}F$, F, $H_{1\text{-}36}$	0		0	
46.96648952357635, 46.970287436423654	21	NS, CS, S, NO_2, CO_2, $O_{1\text{-}2}$, N_2O, NO, CNO, $C_{1\text{-}2}O$, $N_{1\text{-}3}$, CN_2, $C_{1\text{-}2}N$, $C_{1\text{-}3}$	95	$H_{1\text{-}11}Cl$, Cl, $H_{1\text{-}8}F_2$, $F_{1\text{-}2}$, $H_{1\text{-}27}F$, $H_{1\text{-}46}$	1	CCl	1	CCl
48.962558174089345, 48.96840118591066	24	OS, NS, CS, S, $O_{1\text{-}3}$, NO_2, CO_2, N_2O, NO, CNO, $C_{1\text{-}2}O$, $N_{1\text{-}3}$, CN_2, $C_{1\text{-}2}N$, $C_{1\text{-}4}$	103	$H_{1\text{-}13}Cl$, Cl, $H_{1\text{-}10}F_2$, $F_{1\text{-}2}$, $H_{1\text{-}29}F$, $H_{1\text{-}48}$	0		0	

6.2 Faster knapsack: avoiding enumerating fragments of negative DBE value

With two arbitrary lists, many impossible partial fragments are enumerated, in particular with too many hydrogens. It would speed-up the process to know an upper bound on the number of mono-valent atoms. To be able to compute such value, the first list, denoted M, is now made of the multi-valent atoms and the second list, denoted m, made of mono-valent atoms only. In this way, after computing the list M, one can compute the DBE value of each partial fragment made of multi-valent atoms only. An upper bound on the number of mono-valent atoms is two times the maximum DBE value obtained over the list M. This upper bound allows to constraint the enumeration of the list m, hence reducing the running-time and the length of the second list. Table 6 presents a comparison of the lengths of the lists A, B, M and m for the target masses of CCl_4. We observed that the list m is at least two times smaller than the list B.

Table 6 Intermediate lists in the knapsack algorithm with two sets of atoms {C, N, O, S, Br} for the list A and {H, F, Cl, I} for the list B, then with a set of multi-valent atoms {C, N, O, S} giving the list M, and a set of mono-valent atoms {H, F, Cl, Br, I} giving the list m. After enumerating the list M, the maximum possible valence is computed and used as an upper bound on the number of mono-valent atoms to generate the list m. One observes that this technique allows to divide by more than two the length of the second list.

target mass interval	#A	#B	# sol.	#M	2 max DBE	#m	DBE ≥ 0
34.96751070677594, 34.97006625322406	10	51	1	10	6	13	1
35.974137795964566, 35.97778836403543	10	54	1	10	6	14	1
36.96406557296814, 36.967505987031856	11	56	0	11	8	18	0
46.96648952357635, 46.970287436423654	21	95	1	21	8	31	1
48.962558174089345, 48.96840118591066	24	103	0	24	10	39	0
59.960220186945, 59.971315773055004	37	156	1	37	10	48	1
81.9330864132061, 81.9395331467939	90	315	1	89	14	110	1
82.93831758560036, 82.95111397439963	94	324	3	93	14	115	2
83.93024931474109, 83.9372426452589	94	333	0	93	14	117	0
84.92634272134954, 84.97112963865045	101	342	7	100	16	135	4
85.92419323227152, 85.93924312772849	101	351	1	100	16	137	1
97.9236485334006, 97.9389656265994	154	477	3	150	18	189	2
99.90729357057015, 99.94127718942985	161	501	4	157	18	197	3
116.90200574284233, 116.90847941715768	274	735	2	262	20	293	1
117.89455759263474, 117.92205636736527	275	751	5	262	20	300	3
118.89897942315969, 118.9056775368403	287	766	0	274	20	305	0
119.89648859190275, 119.91785116809724	290	782	2	275	20	311	1
120.89523104367791, 120.90302931632209	306	798	1	291	22	345	0
122.8875535007597, 122.90537265924031	323	830	1	305	22	357	1

Table 7 Measured masses at RT = 1708.27s. In blue, the correct guess made by knapsack. In orange, the identified isotopocules.

measured mass (m/z)	mass range with uncertainty	intensity	knapsack	identified
34.96878848	[34.96751070677594, 34.97006625322406]	2722.2042	Cl	Cl
35.97596308	[35.974137795964566, 35.97778836403543]	1051.6898	HCl	HCl
36.96578578	[36.96406557296814, 36.967505987031856]	914.6638	–	$[^{37}Cl]$
46.96838848	[46.96648952357635, 46.970287436423654]	3784.4981	CCl	CCl
48.96547968	[48.962558174089345, 48.96840118591066]	1192.8077	–	$C[^{37}Cl]$
59.96576798	[59.960220186945, 59.971315773055004]	120.657	COS	–
81.93630978	[81.9330864132061, 81.9395331467939]	6160.9695	CCl_2	CCl_2
82.94471578	[82.93831758560036, 82.95111397439963]	319.247	S_2F, $HCCl_2$	$HCCl_2$
83.93374598	[83.93024931474109, 83.9372426452589]	3956.1947	–	$CCl[^{37}Cl]$
84.94873618	[84.92634272134954, 84.97112963865045]	140.2337	H_2S_2F, H_2OSCl, $HNCl_2$, CF_2Cl	$HCCl[^{37}Cl]$
85.93171818	[85.92419323227152, 85.93924312772849]	564.31	OCl_2	$C[^{37}Cl]_2$
97.93130708	[97.9236485334006, 97.9389656265994]	134.1543	H_2S_3, $COCl_2$	$COCl_2$
99.92428538	[99.90729357057015, 99.94127718942985]	106.7792	HS_2Cl, HO_2SCl, $NOCl_2$	$COCl[^{37}Cl]$
116.90524258	[116.90200574284233, 116.90847941715768]	28974.7117	CCl_3	CCl_3
117.90830698	[117.89455759263474, 117.92205636736527]	189.527	S_2FCl, $OSCl_2$, $HCCl_3$	$[^{13}C]Cl_3$
118.90232848	[118.89897942315969, 118.9056775368403]	29078.7276	–	$CCl_2[^{37}Cl]$
119.90716988	[119.89648859190275, 119.91785116809724]	182.0316	C_2S_3	$[13C]Cl_2[^{37}Cl]$
120.89913018	[120.89523104367791, 120.90302931632209]	9220.1959	–	$CCl[^{37}Cl]_2$
122.89646308	[122.8875535007597, 122.90537265924031]	886.6747	CSBr	$C[^{37}Cl]_3$

The chosen possible atoms are H, C, N, O, F, S, Cl, Br, I. At start, only the abundant atoms are considered. The multi-valent atoms are C, N, O, S, the mono-valent are H, F, Cl, Br, I.

Consider now the target mass $m = 116.90524258$ m/z, with uncertainty range $m_{\text{min}} = 116.90200574284233$, $m_{\text{max}} = 116.90847941715768$. Our knapsack algorithm first lists all possible chemical formulae made of any number of the multi-valent atoms and so that the mass of the fragment is at most m_{max}. There are 263 combinations whose DBE ranges from to 2 to 20. For each fragment, its DBE value n is computed and a second knapsack algorithm is run to find a complement fragment made of at most n mono-valent atoms so that the total mass fits within the bounds $m_{\text{min}}, m_{\text{max}}$ and the total DBE is positive or zero. For the mass $m = 116.90524258$ m/z, there is one solution: CCl3.

To account for chemical formulae made of mono-valent atoms only, a final knapsack algorithm is run to find the chemical formulae with only one or two mono-valent atoms whose mass fits in the uncertainty range (this gives the solution Cl for the mass 34.96878848).

Table 8 isotopocules of CCl_4 and relative intensity. See also Fig. 3n the main article.

isotopocule	mass (m/z)	proportion	relative intensity
C	12.00000000	0.988922	1.000000
$[^{13}C]$	13.00335484	0.011078	0.011202
Cl	34.96885271	0.757647	1.000000
$[^{37}Cl]$	36.96590260	0.242353	0.319876
CCl_4	151.87541084	0.325859	1.000000
$CCl_3[^{37}Cl]$	153.87246073	0.416938	1.279504
$CCl_2[^{37}Cl]_2$	155.86951062	0.200052	0.613923
$CCl[^{37}Cl]_3$	157.86656051	0.042661	0.130920
$C[^{37}Cl]_4$	159.86361040	0.003412	0.010470
$[^{13}C]Cl_4$	152.87876568	0.003650	0.011202
$[^{13}C]Cl_3[^{37}Cl]$	154.87581557	0.004671	0.014333
$[^{13}C]Cl_2[^{37}Cl]_2$	156.87286546	0.002241	0.006877
$[^{13}C]Cl[^{37}Cl]_3$	158.86991535	0.000478	0.001467
$[^{13}C][^{37}Cl]_4$	160.86696524	0.000038	0.000117

7 Results for the validation set

Results for the validation set are given in Fig. 4 and Fig. 5.

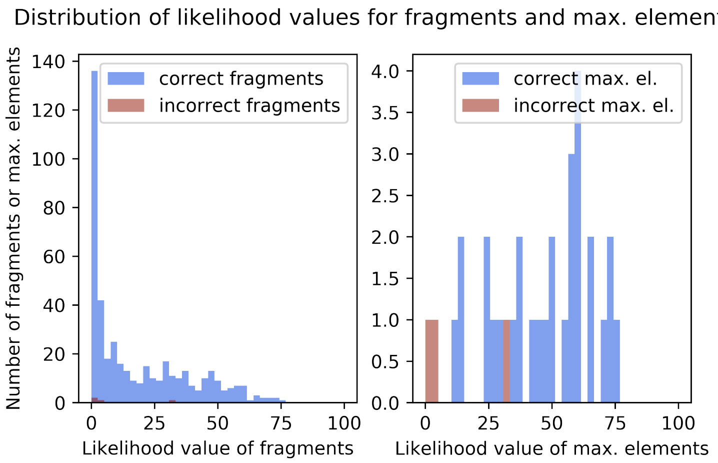

Figure 4 Distribution of likelihood values of fragments (left) and maximal fragments (right) assigned to a measured mass, for the validation set (23 compounds). A likelihood value of 100 indicates that the chemical formula of the fragment or maximal fragment is highly likely. Blue: distribution for correctly identified fragments/maximal fragments assigned to a measured mass. Red: distribution for wrongly identified fragments/maximal fragments.

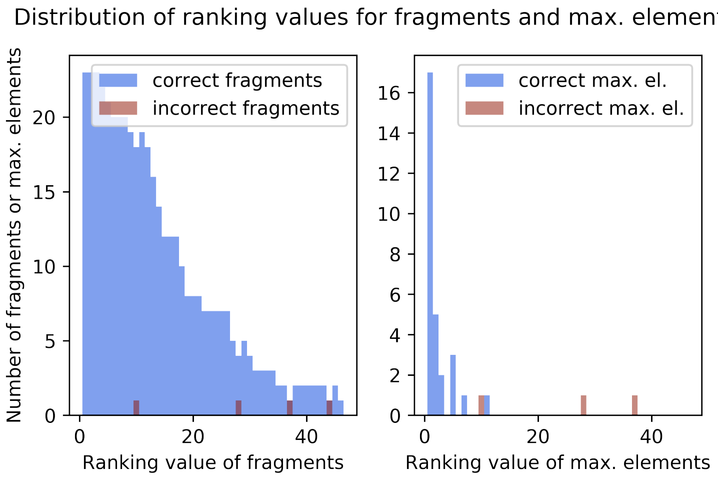

Figure 5 Distribution of ranking values for fragments and maximal fragments assigned to a measured mass, for the validation set (23 compounds). A ranking value of 1 means that the fragment/maximal fragments was ranked as most likely (maximum likelihood value within the set of fragments/maximal fragments). Blue: distribution of ranking for correctly identified fragments/maximal fragments assigned to a measured mass. Red: distribution of ranking for wrongly identified fragments/maximal fragments.

8 Testing the MOLGEN-MS commercial software

MOLGEN-MS is a commercial software aiming at reconstucting the correct chemical formula and structure of an unknown substance for which the EI spectrum is provided, with unit mass resolution. The MOLGEN-MS workflow is made of the following steps [5]:

- MSclass: module to identify which chemical classes are likely present or not in the EI mass spectrum. This module uses a pre-defined list;
- ElCoCo: module to guess the most likely weight(s) of the molecular ion, with unit mass resolution. It can (or not) uses inputs form MSclass. Using the guessed molecular weight(s), all matching chemical formulae are generated, using a list of chemical elements that can be user-defined.
- MOLGEN: using all generated chemical formulae, generates all possible chemical strucutures. These structures are then fragmented and the obtained fragments are compared to the measured ones. All candidate structures are ranked, the most likely first.

We used a 90-days free version kindly provided by Markus Meringer, version 1.0.1.5. We first converted all our data into a compatible format (.TRA). All masses were converted to unit mass resolution. We used the default settings in MOLGEN-MS and keeping user intervention at the minimum. We used the same chemical elements as in our software: H, C, N, O, F, S, Cl, Br, I. We do not use Si and P that occur very seldom in our type of samples. We tested MOLGEN-MS using two different settings: with and without the MSclass module. When using the MSclass module, the output was then directly used in the ElCoCo module, without any user manipulation of the results. For the ElCoCo module, we used the default parameter for the confidence interval for guessing the molecular weight (98). We did not use a minimum match value to eliminate candidate formula with a match value lower than a threshold. We then checked if the correct molecular weight was listed within the solutions.

We do not include the following substances in the discussion below because their true molecular wieght is outside our detection mass range: C_6F_{14} in the training set and TCHFB in the validation set.

In MOLGEN-MS, when using MSclass followed by ElCoCo, on the training set, only three substances have the correct molecular formula listed (COS, benzene and dichlorodifluoromethane). We actually obtain better results when not using the submodule MS-class. When using ElCoCo alone, with the default precision value for the molecular weight (98), and no minimum precision for the molecular formula, the correct molecular formula is listed in 15 cases of the training set (out of 35, this is 43%), with rankings from 1 to 45. If a user manually puts in the correct molecular weight, then the correct molecular formula is listed in 28 cases (out of 35, this is 80%). Two cases with no correct solution require the Sulphur atom to have a set valence of 6, which may not be the default setting in MOLGEN-MS. In 6 cases, the correct molecular formula was listed only if the correct number of each atom was given as input. We do not have an explanation for this.

MOLGEN-MS provides better results on the validation set: for 17 compounds out of 22 (77%), the correct molecular formula is listed (without using MSclass), with ranking from 1 to 46. On average, the correct molecular formula is listed using

MOLGEN-MS-ElCoCo in 56% of the cases, in the worse conditions where no user intervention is provided at all.

We provide all input data file in a MOLGEN-MS compatible format as zip file.

We believe that to identify the structure of an unknown compound, a promising setup may be to first run our ALPINAC software on the high resolution mass spectrum, and use the suggested molecular ion(s) together with the unit mass resolution spectrum as input to MOLGEN-MS, where the MOLGEN module would be used.

Author details

[1]Laboratory for Air Pollution /Environmental Technology, Empa, Swiss Federal Laboratories for Materials Science and Technology, Ueberlandstrasse 129, 8600 Dübendorf, Switzerland. [2]Université de Lorraine, CNRS, Inria, LORIA, 54000 Nancy, France.

References

1. NIST: NIST EPA NIH Mass Spectral Library. Online database (2020). https://www.nist.gov/srd/nist-standard-reference-database-1a-v17 Accessed 11.03.2020
2. ChemSpider: ChemSpider: Search and share chemistry. Online database (2020). https://www.chemspider.com Accessed 11.03.2020
3. National Center for Biotechnology Information: Online database of the National Library of Medicine. Online (2020). https://pubchem.ncbi.nlm.nih.gov/ Accessed 08.08.2020
4. Yergey, J.A.: A general approach to calculating isotopic distributions for mass spectrometry. International Journal of Mass Spectrometry and Ion Physics **52**, 337–349 (1983). doi:10.1002/jms.4498. e4498 JMS-20-0003
5. Meringer, M.: MOLGEN-MS: Evaluation of low resolution electron impact mass spectra without database search. Technical report (2000). https://molgen.de/documents/MolgenMS_manual/index.html
